# Thermal Depolymerization Challenges of PTFE:Silicone Rubber Mixtures and Composite Materials

**DOI:** 10.3390/polym18151929

**Published:** 2026-08-06

**Authors:** Lukas Eigenschink, Matthias Mastalir, Michael Harasek, Christian Paulik

**Affiliations:** 1 Institute for Chemical Technology of Organic Materials, Johannes Kepler University Linz, Altenberger Straße 69, 4040 Linz, Austria; christian.paulik@jku.at; 2 Institute of Chemical, Environmental and Bioscience Engineering, TU Wien, Getreidemarkt 9/166, 1060 Vienna, Austria; michael.harasek@tuwien.ac.at; 3 OMV Downstream GmbH, Mannswörther Straße 28, 2320 Schwechat, Austria; matthias.mastalir@omv.com

**Keywords:** silicone rubber, PTFE, PFAS, co-pyrolysis, thermal recycling, depolymerization

## Abstract

Thermochemical depolymerization of polymer mixtures and composite materials is challenging due to non-additive degradation behavior and the emergence of new reaction pathways during pyrolysis. Both polytetrafluoroethylene (PTFE) and silicone rubber (SR) can depolymerize into monomers or low-molecular-weight oligomers when pyrolyzed individually, making them, in principle, suitable candidates for depolymerization-based recycling. Because they are frequently combined in technical applications and composites, their behavior during co-pyrolysis warrants investigation. However, the pyrolysis of PTFE:SR mixtures and composites remains poorly understood. In this study, we examine the pyrolysis behavior of PTFE:SR systems with emphasis on mass balance, product composition, and the formation of new species to address potential limitations for depolymerization-based recycling. Experiments were conducted on virgin PTFE and SR, defined polymer mixtures, and commercially relevant composites, including PTFE-lined silicone tubing and PTFE:SR septa. The results reveal a pronounced, non-linear dependence of product distribution on PTFE content. Product identification by GC-MS, NMR, and FTIR indicates cleavage of Si–O and Si–CH_3_ bonds and the formation of fluorinated siloxanes as well as new per- and polyfluoroalkyl substances (PFAS). At low PTFE contents, liquid products are dominated by cyclic siloxanes (D_x_). These findings show that depolymerization strategies developed for pure polymers cannot be directly applied to PTFE:SR composites. While systems with low PTFE content may be more amenable to depolymerization, higher PTFE fractions promote the formation of PFAS and difficult-to-valorize fluorinated silicon species. These products complicate selective monomer recovery and could pose significant challenges for the depolymerization-based recycling of PTFE-rich composites.

## 1. Introduction

The rapid expansion of global plastic production has made plastic waste one of the most significant environmental challenges of the twenty-first century. With an annual output exceeding 400 million metric tons [1], a substantial proportion of post-consumer plastics ultimately accumulates in landfills, is incinerated, or escapes into natural ecosystems, where it contributes to pollution, greenhouse-gas emissions, and ecological degradation [2]. Conventional mechanical recycling, although widely implemented, is fundamentally limited by the heterogeneous nature of plastic waste streams, contamination issues, and the progressive deterioration of material quality over multiple recycling cycles [3]. These constraints have intensified interest in advanced recycling approaches capable of processing mixed and compositionally complex waste. Among such strategies, pyrolysis has emerged as a particularly promising route for converting plastic waste into fuels, chemical feedstocks, and reusable molecular building blocks [4]. Pyrolysis involves the thermal decomposition of polymers in the absence of oxygen, leading to the formation of gaseous, liquid, solid products and residue. The composition of these products depends on a variety of factors, including polymer type, temperature, heating rate, and residence time [5]. In contrast to mechanical recycling, pyrolysis is inherently suited for mixed plastic waste [6]. Nevertheless, real waste streams typically consist of complex polymer mixtures [7] whose thermal decomposition behavior in most cases cannot be predicted by simply extrapolating from single-polymer studies. Interactions between polymers during thermal conversion may result in altered degradation pathways, changes in product selectivity, or the formation of new compounds [8,9,10]. Despite the practical relevance of such phenomena, synergistic effects in mixed-polymer pyrolysis remain insufficiently understood.

Within this context, polytetrafluoroethylene (PTFE) and silicone rubbers (SR), primarily polydimethylsiloxane-based [11,12,13], have attracted increasing academic interest regarding their thermal conversion behavior [14]. Silicone materials depolymerize predominantly via a six-membered transition state into cyclic siloxanes [15], which represent valuable intermediates for the synthesis of new silicone-based materials [16,17,18]. PTFE, in contrast, degrades via random chain scission. Subsequent α-scission followed by radical recombination, together with β-scission, yields the monomer tetrafluoroethylene in large quantities, which is suitable for fluoropolymer synthesis, together with minor amounts of other fluorocarbon species [19,20]. Thus, thermal treatment of both polymer classes offers opportunities not only for waste volume reduction but also for chemical feedstock recovery.

Co-pyrolysis of mixed plastics has gained increasing interest in both academia and industry due to the inherent heterogenous nature of plastic waste streams [7,21,22]. The available literature remains limited compared to studies on single-polymer pyrolysis. Numerous investigations using different polymer mixtures have demonstrated that interactions between different polymers can influence degradation kinetics [8,23,24], promote or suppress specific reaction pathways [25,26], and alter both product yields and product composition [27,28]. These effects are generally attributed to interactions between reactive degradation intermediates and radical species originating from the individual polymers [29,30,31].

Of particular interest are compounds classified as per- and polyfluoroalkyl substances (PFAS). According to OECD, this class of compounds consists of compounds containing one fully fluorinated carbon atom (CF_2_ and CF_3_) [32]. Their overall properties vary significantly; however, many PFAS are regarded as bioaccumulative and toxic [33,34]. To date, studies investigating the fate of PFAS during pyrolysis are scarce.

The investigation of PTFE:SR co-pyrolysis is motivated by their frequent co-occurrence in real waste streams. Both materials share key functional properties, including high thermal stability, chemical inertness and excellent electrical insulation. As a result, they are commonly used together in similar application areas such as sealing and gasket systems, electrical and electronic components, medical and laboratory devices, food processing equipment and automotive or aerospace technologies. Typical examples include valve assemblies combining PTFE seats with silicone O-rings, septa, cable systems featuring PTFE insulation with silicone encapsulation, and fluid-handling components consisting of silicone tubing with PTFE liners. The multi-material nature of these products often complicates separation at end-of-life, leading to mixed silicone–fluoropolymer fractions in industrial maintenance waste, electronic waste and dismantled equipment streams [35,36].

Simultaneous thermal conversion introduces the possibility of interactions between silicon-containing and fluorine-containing reactive degradation intermediates, potentially influencing reaction mechanisms, product distributions and overall conversion pathways. Understanding these synergistic effects is essential for accurate process modeling and evaluation of chemical recycling strategies for mixed polymer waste.

Therefore, studying the co-pyrolysis of SR and PTFE addresses both a fundamental scientific gap and a practical challenge regarding recycling and depolymerization strategies. From a technological perspective, it reflects realistic mixed-polymer scenarios encountered in high-performance applications. From a chemical perspective, it provides insight into interactions between two thermally robust polymer systems whose degradation products possess intrinsic value as chemical building blocks. Advancing the understanding of synergistic effects in PTFE:SR co-pyrolysis may support the development of targeted thermal recycling processes, improve resource recovery from complex technical plastic waste streams and enable novel synthesis pathways based on mixed polymer feedstocks.

## 2. Materials and Methods

### 2.1. Materials

Polydimethylsiloxane-based (PDMS) silicone rubber (product type: Genioplast ^®^ Pellet S) was provided as opaque pellets containing approximately 70 wt% siloxane polymer and 30 wt% fumed silica (SR-V) by Wacker Chemie AG (Munich, Germany). PTFE (product type: KT-300M) was supplied by Kitamaru Limited (Togo-cho, Japan) as a white, additive-free powder (PTFE-V). In addition to the pure polymers, SR- and PTFE-based tubing (SR-T and PTFE-T) as well as commercially relevant PTFE and SR composites, namely PTFE-SR septum and PTFE-lined SR tubing (PTFE-SR-T), were examined, all provided by VWR International (Radnor, PA, USA). Mechanical separation and weighing of the PTFE-lined tubing showed that the PTFE liner accounted for 5 wt% of the total tube mass. For PTFE-SR septum, the PTFE fraction accounted for approximately 13 wt%. For polymer mixtures, the polymers were ground, physically combined and mixed by repeated stirring. Composite materials were reduced in size by cutting in order to preserve the original composite architecture during subsequent experiments.

### 2.2. Thermogravimetric Analysis (TGA)

Thermogravimetric analysis (TGA) of the polymers, their mixtures and composite materials were performed using a Perkin Elmer—TGA 8000 (Shelton, CT, USA) at a temperature range from 25–800 °C (heating rate of 10 °C min^−1^) under a N_2_-atmosphere. Additional experiments under the same conditions with an isothermal hold at 400 °C for 30 min were also conducted. An important parameter for TGA is the peak degradation temperature (T_P_), which is defined as the temperature at which the maximum rate of mass loss occurs. The onset temperature (T_O_) and end temperature (T_E_) were defined as the points corresponding to 1% of the maximum mass-loss rate before and after the peak temperature (T_P_). The degradation interval (ΔT) was calculated as the difference between T_E_ and T_O_.

### 2.3. Laboratory Pyrolysis

The pyrolysis experiments were conducted in a static nitrogen atmosphere using a laboratory-scale batch reactor assembled from standard glassware. Prior to each experiment, the reactor was purged by five evacuations (3 min)/nitrogen refill cycles and subsequently operated under a static nitrogen atmosphere to enable interactions between PTFE- and silicone-derived intermediates. Under these static conditions, volatile degradation products remain in the reactor until condensation, resulting in long vapor residence times that could promote secondary gas-phase reactions as well as recombination or condensation at colder surfaces. Because all experiments were conducted using the same reactor geometry and identical operating parameters, the data are intended for relative comparison between pure polymers, mixtures, and composites. For the pyrolysis, 5 g of pure polymers, mixtures and composite materials were used. Pyrolysis of each pure polymer and composite was conducted in triplicate, while polymer mixtures were tested twice to support reproducibility. The starting material was placed in a round bottom flask (flask 1) and heated using a high-power external thermal source applied directly to the wall of flask 1. The reactor was heated externally to a maximum of 600 °C, measured at the outer wall of flask 1, using an infrared pyrometer. Because the temperature was measured at the reactor wall rather than within the reaction zone, the actual sample temperature may lag behind the indicated value. This limitation should be considered when interpreting pyrolysis temperature and product formation. However, all experiments were conducted under identical heating conditions. The heating rate was calculated to be approximately 30 °C s^−1^. The temperature was held for 10 min, after which further gas production was not observed. The residue fraction remained in flask 1, whereas the evolved gas was cooled through a distillation condenser (20 °C) where the condensed liquid and solid products were collected in another cooled (20 °C) round bottom flask (flask 2). The gaseous products, which are non-condensable at 20 °C, were collected in a gas sample bag. For mass balancing, the yield of the gaseous fraction was calculated from the difference between the mass of the sample and the liquid/solid products and the residue fraction. Liquid and solid products were separated by washing with dichloromethane (2 × 5 mL). The dichloromethane-soluble fraction was subjected to GC–MS analysis, while the insoluble solid fraction was characterized by FTIR spectroscopy. The collected products were stored under a N_2_-atmosphere until further analysis. To evaluate deviations from the mass balance in polymer mixtures, calculated mass fractions were determined. The calculated mass fraction of the liquid, gaseous and residue products ($x_{l,g,r}^{calc}$) were determined from the mass fraction of each polymer in the feed $m_{i}$ and the corresponding product mass fractions obtained from the pure polymers ($x_{l,g,r}^{i}$) using Equation (1).

(1) $$x_{l,g,r}^{calc}=m_{PTFE}\times x_{l,g,r}^{PTFE}+\;m_{SR}\times x_{l,g,r}^{SR}$$

### 2.4. Gas Chromatography—Mass Spectrometry (GC-MS)

The isolated liquid products were diluted with dichloromethane and passed through a small layer of CaCO_3_ as a precautionary measure to protect the GC-MS system from potential traces of hydrogen fluoride before analysis. The products were separated using a gas chromatograph (Thermo Finnigan Trace GC; San Jose, CA, USA) equipped with a capillary column (Rtx-5ms 5% diphenyl, 95% dimethylpolysiloxane, 30 m × 0.25 mm inner diameter × 0.25 µm film thickness) using a 1.5 mL min^−1^ Helium flow. Samples were introduced through the inlet (280 °C, split flow 50 mL min^−1^, split ratio 33) and separated using a temperature program starting at 40 °C held for 1 min and followed by a heating ramp of 15 °C min^−1^ to 300 °C, which was held for 2 min. The mass spectra were recorded using a PolarisQ iontrap mass spectrometer (Thermo Finnigan; San Jose, CA, USA) under 70 eV electron ionization in a *m*/*z* range of 25–800 at 1.96 scans s^−1^. The compounds detected through GC-MS were identified using NIST-library [37], Wiley database [38], reference substances, the literature data or through manual interpretation of the mass spectra.

For the analysis of the gaseous phase, GC-MS was employed. The products were separated using a gas chromatograph (Agilent 8890 GC; Santa Clara, CA, USA) equipped with a GS-Gaspro column (60 m × 0.32 mm inner diameter; Santa Clara, CA, USA). The sample was separated using a temperature program starting at 43 °C held for 5 min and followed by a heating ramp of 10 °C min^−1^ to 200 °C, which was held for 7 min. Mass spectra were recorded using an Agilent 5977B Mass Spectrometer (Santa Clara, CA, USA) under 70 eV electron ionization in a *m*/*z* range from 10–300. The compounds were identified using the GC-MS Enhanced MassHunter software (version 13.0) and by comparison with the NIST-library [37], Wiley database [38] and the literature data. All GC-MS results are reported as normalized peak area percentages to 100% and should be regarded as semi-quantitative. Owing to the differing ionization efficiencies and detector response factors of the various compound classes, the reported peak areas do not represent absolute concentrations or product yields. Instead, they are used to compare relative changes in product composition between experiments performed under identical conditions.

### 2.5. Fourier Transform Infrared Spectroscopy (FTIR)

Infrared spectra were recorded in attenuated total reflection mode using a Perkin Elmer Spectrum Two FT-IR spectrometer (Shelton, CT, USA) in a wavenumber interval from 4000–400 cm^−1^.

### 2.6. Nuclear Magnetic Resonance Spectroscopy (NMR)

NMR spectra were recorded using a Bruker Avance 400 (400 MHz; Bruker BioSpin GmbH, Rheinstetten, Germany) in CDCl_3_. CDCl_3_ was stored over silver foil and filtered through a short plug of basic alumina just before use. All chemical shifts are reported in ppm and were externally referenced to CFCl_3_ for ^19^F-NMR (−220–−20 ppm) and Si(CH_3_)_4_ for ^29^Si-NMR (−350–−150 ppm).

## 3. Results and Discussion

This study investigates the pyrolysis behavior of PTFE and SR, with emphasis on mass balance, product composition, and product distribution to identify synergistic effects and assess depolymerization. To capture both idealized and application-relevant systems, three material classes and their corresponding mixtures were investigated: virgin polymers (PTFE-V and SR-V), tubing materials composed of silicone rubber (SR-T) and PTFE (PTFE-T), and commercially relevant composite materials, specifically a PTFE-SR septum and PTFE-lined silicone rubber tubing (PTFE-SR-T). TGA experiments were conducted to investigate possible stepwise depolymerization. Laboratory-scale pyrolysis experiments were conducted on the individual polymers as well as co-pyrolysis experiments using polymer mixtures. In this study, the term co-pyrolysis refers to PTFE-V:SR-V mixtures, PTFE-T:SR-T mixtures and composite materials tested, if not stated otherwise. Throughout this manuscript, PTFE:SR is used as a general notation for co-pyrolysis systems consisting of polytetrafluoroethylene and silicone rubber, including polymer mixtures and composite materials unless stated otherwise. Specific material types are distinguished by additional identifiers: PTFE-V:SR-V refers to virgin polymer mixtures, PTFE-T:SR-V denotes mixtures prepared from silicone and PTFE tubing materials, and hyphenated nomenclature is used for commercially manufactured composite materials, where both polymers are physically integrated (e.g., PTFE-SR-T for PTFE-lined silicone tubing and PTFE-SR septum for the composite septum material).

The resulting products were systematically separated and characterized: solid residues were analyzed using FTIR and gaseous products and liquid fractions were analyzed via GC-MS while liquid product analysis was supported by NMR analysis. To ensure structural assignment, particular attention was directed toward characterizing the fragmentation behavior of the newly formed species, which often lack available references. Based on the identified pyrolysis products, a mechanistic framework is proposed to rationalize the observed product distributions and possible interactions between PTFE- and silicone-derived degradation intermediates.

### 3.1. Limitations on Thermal Separation

**Figure 1:**
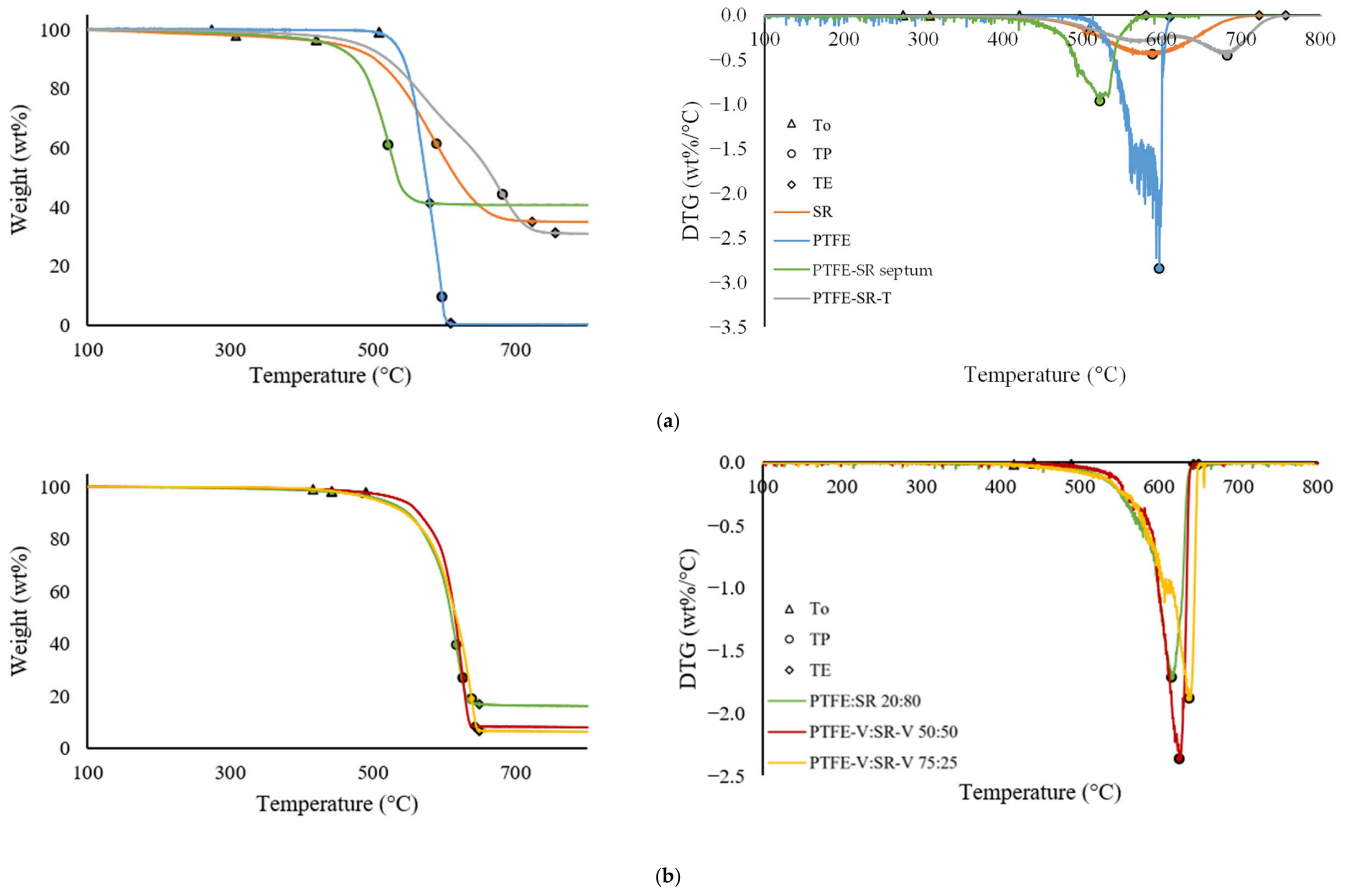
TGA and DTG curves of (**a**) SR-V, PTFE-V, and PTFE:SR composites and (**b**) PTFE-V:SR-V mixtures. T_P_ = peak degradation temperature; T_O_ and T_E_ = temperatures corresponding to 1% of the maximum weight-loss rate before and after T_P_, respectively; ΔT = T_E_ − T_O_.

Selective depolymerization based on stepwise thermal treatment is proposed as a potential recycling strategy for dissimilar mixed polymer systems when their thermal degradation temperatures appear sufficiently separated [39,40,41]. The feasibility of such an approach was evaluated for PTFE-V and SR-V by TGA of the individual polymers, mixtures, and the PTFE-SR septum, as shown in Figure 1. The parameters for the TGA are summarized in Table 1. While the onset of SR degradation occurs at lower temperatures than that of PTFE, SR decomposition proceeds over a comparatively broad temperature range. The TGA and DTG data of the PTFE–SR mixtures and composite materials exhibit a single, broad mass-loss event rather than two discrete degradation steps.

Due to the comparable low onset temperature of SR, the TGA curves indicate the existence of a narrow temperature region around approximately 400 °C in which SR degradation could, in theory, take place prior to extensive PTFE decomposition. Such a window might suggest the possibility of selectively depolymerizing SR while leaving PTFE largely unaffected, thereby enabling sequential product recovery. However, the experimental results of this study demonstrate that this apparent thermal separability does not translate into practical process selectivity. Even under isothermal conditions at 400 °C for 30 min, two distinct degradation steps could not be observed (Figure S1). Consequently, both dynamic heating and isothermal holding at intermediate temperatures lead to overlapping degradation rather than isolated depolymerization of a single component. The apparent continuity of the degradation profile is most likely attributable to the substantial overlap of the broad SR degradation interval with degradation of PTFE. Nevertheless, additional factors cannot be excluded. Interactions between reactive degradation intermediates, radical-mediated initiation reactions, and changes in heat and mass transfer resulting from the intimate mixing of the polymers may also contribute to the observed behavior. This interpretation is supported by the observation that mixtures with high PTFE content (75 wt%) lower the overall onset temperature compared to mixtures with lower PTFE content. This behavior suggests that factors beyond a simple overlap of the degradation intervals may be involved. Nevertheless, these findings clearly indicate that stepwise pyrolysis based solely on temperature control is not feasible for PTFE:SR systems under the tested conditions, necessitating a mechanistic investigation of their co-pyrolysis behavior. Consequently, the following sections investigate the interactions occurring during their simultaneous thermal degradation. A pyrolysis temperature of 600 °C was therefore selected, as it is slightly above the peak degradation temperatures of both polymers, promoting concurrent degradation and thereby enabling potential co-pyrolysis interactions to be investigated.

### 3.2. Single Polymer Pyrolysis

To assess synergistic effects during co-pyrolysis, SR and PTFE were subjected to individual polymer pyrolysis three times. These individual yield profiles were used to calculate the expected product distribution in the co-pyrolyzed mixtures. During pyrolysis, SR predominantly forms a liquid fraction composed of cyclic siloxanes of various ring sizes, along with a substantial residue arising from its high SiO_2_ filler content as well as residue formation due to decomposition within the PDMS backbone [42]. Its gaseous output is minimal (≈3.6 wt%), consisting mainly of methane, hydrogen, and short-chain hydrocarbons generated through side-chain cleavage and recombination reactions. In contrast, PTFE exhibits almost the opposite behavior: it decomposes primarily into gaseous products (≈96.9 wt%) dominated by tetrafluoroethylene (TFE), octafluorocyclobutane (OFCB), and minor amounts of hexafluoropropene (HFP). Only trace amounts of solid residues are produced, including a fine white powder identified as low-molecular-weight oligomeric PTFE by FTIR. The mass balancing of the individual polymer pyrolysis experiments is summarized in Table 2.

### 3.3. Co-Pyrolysis of PTFE and SR

The mass balance of the PTFE:SR mixtures was strongly influenced by the ratio of the polymers and deviated substantially from the theoretically calculated yields. The observed behavior was similar for both the virgin polymer mixtures and the commercial tubing-based mixtures. Increasing the PTFE content resulted in a pronounced increase in the liquid yield accompanied by a reduction in gaseous products. Because the gaseous fraction of the individual polymers is predominantly derived from PTFE pyrolysis, the observed decrease in gas formation during co-pyrolysis could be attributed to reactions between PTFE-derived intermediates and species originating from silicone degradation. This interaction led to the formation of higher-molecular, condensed products, thereby increasing the liquid yield at the expense of gas formation.

**Figure 2:**
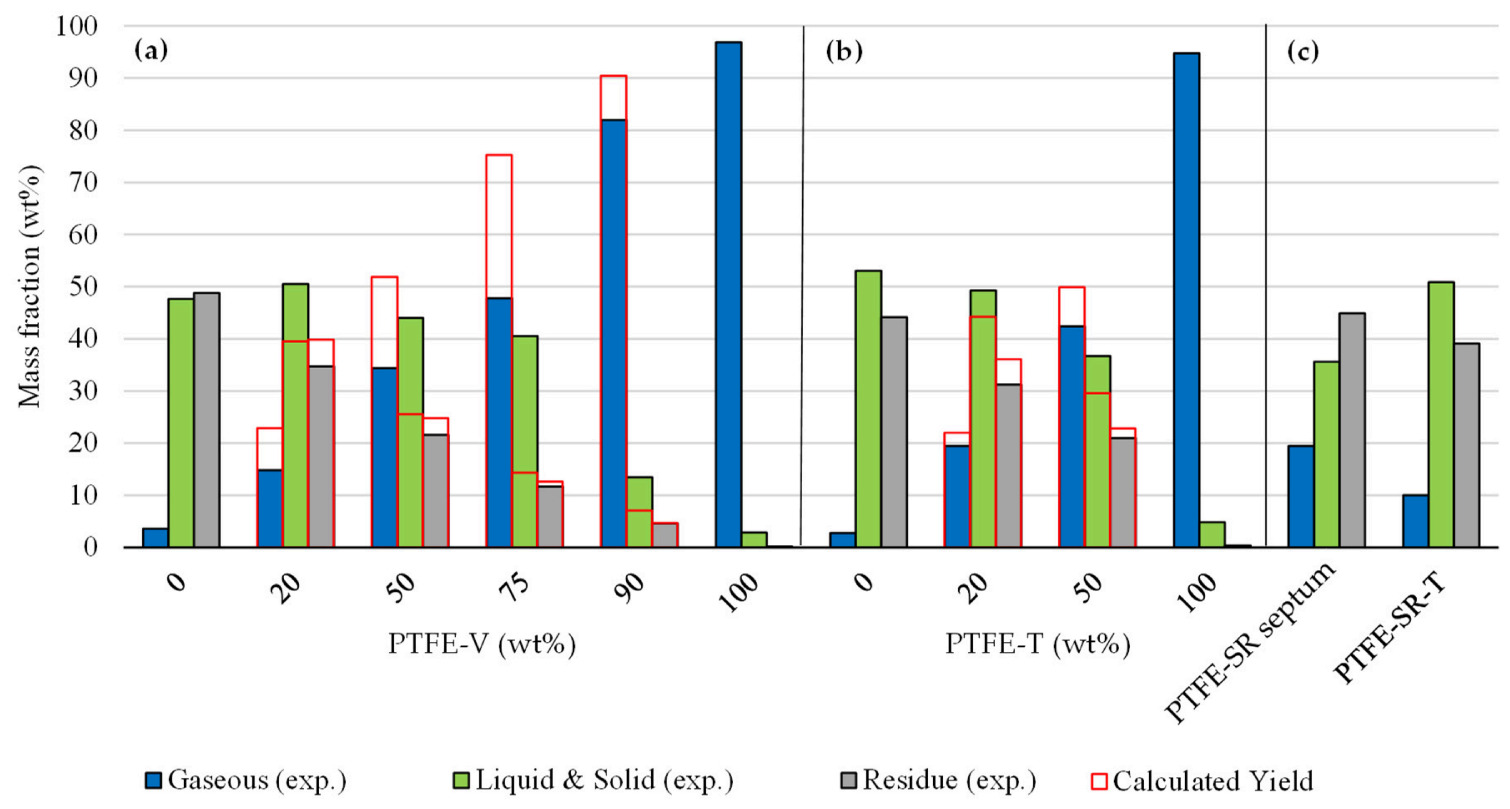
Experimental product yields obtained from the pyrolysis of (**a**) PTFE-V:SR-V mixtures, (**b**) PTFE-T:SR-T mixtures, and (**c**) composite materials. The red line represents the calculated yields based on the pure-polymer pyrolysis data and Equation (1).

Interestingly, the amount of solid residue obtained from the mixtures was lower than the theoretical residue yield. As pure PTFE pyrolysis produces only negligible residue, this reduction suggests that intermediates generated from PTFE interfere with or suppress the formation of silicon-rich residues that are typically produced during silicone degradation. Because residue formation in silicone pyrolysis is primarily attributed to side-chain reactions and cross-linking processes [43], the presence of PTFE-derived radicals may inhibit or disrupt these pathways. The mass balance therefore suggests strong chemical interactions between the respective pyrolysis intermediates and implies a significant modification of the degradation mechanism under co-pyrolysis conditions. Comparable phenomena have been reported for other polymer combinations, where the high reactivity of PTFE-derived radicals could markedly alter decomposition pathways [44]. The liquid co-pyrolysis products exhibited a more intense yellow coloration and appeared predominantly liquid, containing only small suspended solid particles. This contrasts with the mostly crystalline structures commonly observed for the pyrolysis products obtained from pure SR pyrolysis, resulting from the formation of hexamethylcyclotrisiloxane. This suggests substantial changes in the relative product distribution and molecular structure due to interaction with PTFE-derived species. Figure 2 presents the mass fractions of the gaseous, liquid, and residue fractions formed during pyrolysis of pure polymers, mixtures and composites. The calculated yields were determined from the arithmetic mean of the mass-balance data derived from the pure polymers via Equation (1). 

### 3.4. Analysis of Gaseous Products

The gaseous products formed during PTFE pyrolysis were dominated by tetrafluoroethylene (TFE), octafluorocyclobutane (OFCB) and hexafluoropropene (HFP). These products arise from cleavage of the polymer backbone to form CF_2_ radicals, followed by their recombination and β-elimination, in agreement with the literature [19]. Additionally, minor amounts of perfluorinated compounds were detected indicating further recombination of CF_2_ radicals under investigated conditions. In contrast, as demonstrated in previous studies, SR primarily degraded into methane, various short-chain hydrocarbons (CH_4_ ≈ 83 wt%; C_2_H_x_ ≈ 6 wt%), originating from Si–C bond cleavage and side-chain elimination reactions, and hydrogen (H_2_ ≈ 8 wt%) [45,46]. During co-pyrolysis, SR-derived gaseous products were detected only in minor quantities: mainly methane, ethane, and trace short-chain hydrocarbons. This observation can be attributed to the disproportionately large contribution of PTFE-derived volatiles (≈97 wt%), which effectively masks the comparatively small fraction of gaseous products formed from SR (≈3.6 wt%).

Notably, the distribution of PTFE-derived species shifts toward a higher relative abundance of TFE under co-pyrolysis conditions. This behavior is consistent with modified radical reaction pathways: the presence of SR-derived species both dilutes CF_2_ radicals and may promote their interaction with hydrocarbon- and silicon-containing intermediates. Consequently, the effective concentration of free CF_2_ radicals available for recombination may be reduced, suppressing the formation of higher fluorocarbons. As a result, the relative proportion of OFCB and HFP decreases. Consistent with this interpretation, higher molecular weight fluorocarbons observed during pure PTFE pyrolysis are no longer detected under co-pyrolysis conditions, potentially due to competing reactions. Interestingly, the relative proportion of methane, the primary product of SR pyrolysis, decreases with increasing PTFE content compared to other short-chain hydrocarbons. This trend could be attributed to changes in the underlying reaction mechanism. During SR pyrolysis, methane is primarily formed via Si–C bond cleavage followed by hydrogen abstraction from the polymer backbone [45]. Under co-pyrolysis conditions, however, competing hydrogen abstraction by difluorocarbene radicals may partially inhibit methane formation. This could promote a slight increase in the formation of other short-chain hydrocarbons like ethane.

In contrast, a significant number of compounds are formed exclusively during co-pyrolysis. Major compounds were assigned using library matches and published reference spectra, whereas minor byproducts were inferred from manual evaluation of their mass-spectral fragmentation patterns. Because these identifications rely solely on fragmentation analysis, the assignments of the minor species should be regarded as tentative. All assigned products, characteristic ions during fragmentation and their specific rationale for each assignment are summarized in Table S2. In addition to the characteristic decomposition products of the individual components, co-pyrolysis generates a variety of compounds that are not observed during the pyrolysis of pure PTFE or SR. Several fluorine-containing hydrocarbons (e.g., pentafluoroethane, 1,1,2,2-tetrafluoroethane and related species) were formed in the gaseous products. The occurrence of these unique species indicates interactions between fluorocarbon fragments and hydrocarbon- and silicon-containing intermediates in the gas phase. However, at elevated PTFE contents, the abundance of co-pyrolysis-specific products declines. This behavior is likely due to the reduced availability of reactive SR-derived radicals, which diminishes cross-reaction pathways and allows the PTFE degradation mechanism to proceed more independently.

**Figure 3:**
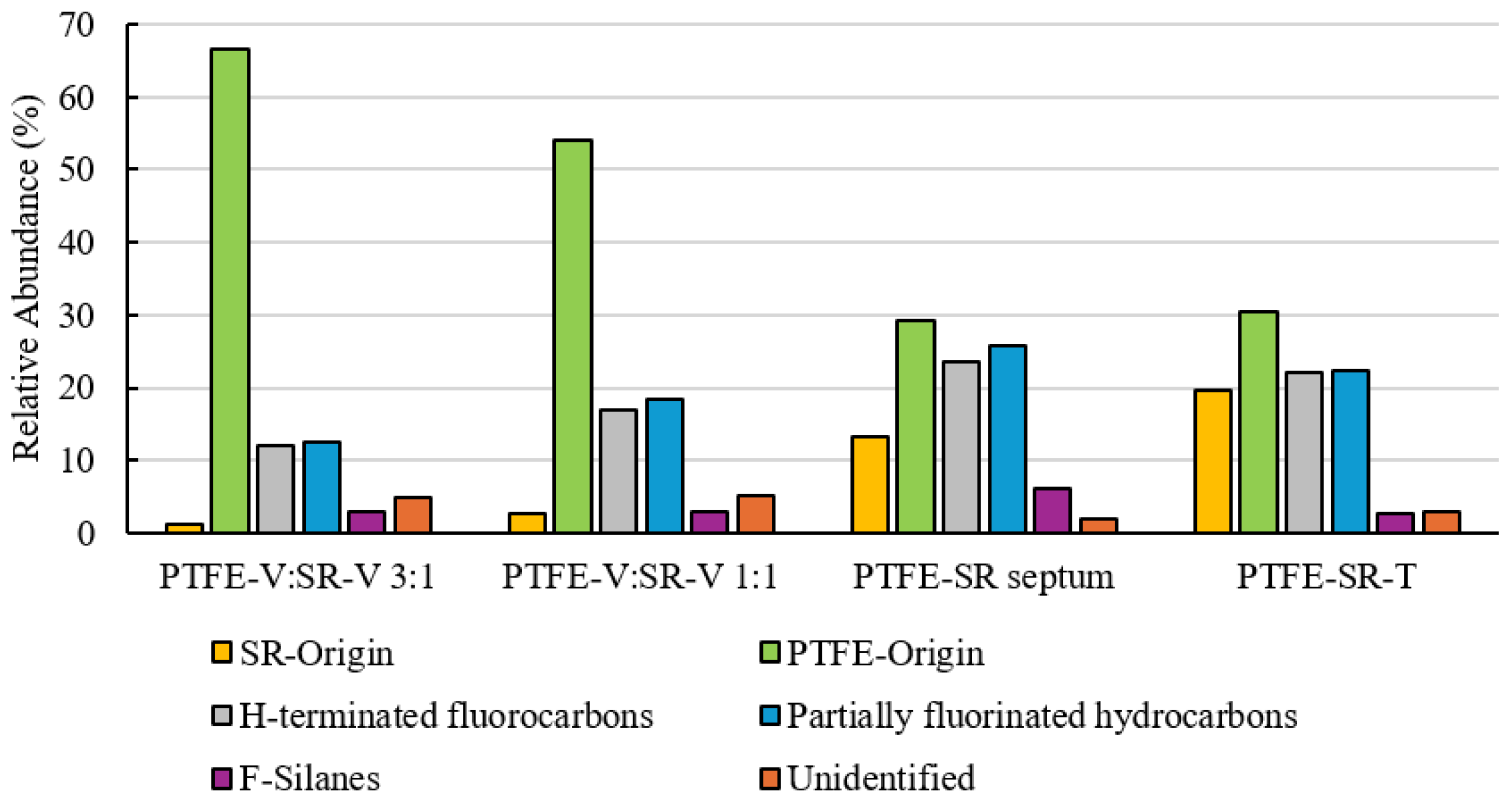
Gaseous products formed during PTFE:SR co-pyrolysis, grouped by product class and assigned polymer origin (silicone rubber or PTFE) based on GC–MS analysis. Compound identification was performed using spectral library matching and, where necessary, manual interpretation of mass spectral fragmentation patterns.

The assigned co-pyrolysis-specific products can be categorized into hydrogen-terminated fluorocarbons, partially fluorinated hydrocarbons (containing multiple hydrogen atoms), and fluorinated silanes. Various hydrogen-terminated fluorocarbons were identified in the condensed phase, covering a chain length range from C_1_ to C_5_. The hydrogen-terminated fluorocarbons most likely emerged through recombination of difluorocarbene radicals and subsequent abstraction of hydrogen from the SR backbone or from intermediates formed during SR degradation. Termination occurs probably either via further hydrogen abstraction, yielding hydrogen-terminated fluorocarbons on both chain ends, or through disproportionation reactions, which produce CF_3_- and CF_2_-terminated species. Furthermore, hydrogen abstraction pathways leading to hydrogen-terminated fluorocarbons could provide a mechanistic explanation for the reduced formation of fully perfluorinated compounds under co-pyrolysis conditions, as hydrogen abstraction is a competing mechanism to perfluorocarbon formation. Partially fluorinated hydrocarbons (e.g., 1,1,2-trifluoropropene) are likely formed through the reaction of difluorocarbene radicals with radical fragments generated during silicone pyrolysis (e.g., methyl radicals) or through Si-C bond cleavage forming mixed fluorinated hydrocarbon structures. Fluorinated silanes, mainly trimethylfluorosilane, are consistent with interactions between the polymers during co-pyrolysis. These findings may be compatible with cleavage of Si-O bonds and the concurrent formation of Si-F bonds, as well as methyl substitution by a fluorine atom. Importantly, tetrafluorosilane (SiF_4_) was not detected under the applied analytical conditions. Together with the consistent methyl substitution observed for the silicon-containing degradation products, this suggests that the detected volatile silicon species originate predominantly from the silicone polymer backbone. However, because highly volatile fluorinated silicon compounds may undergo secondary reactions, adsorption, or analytical losses before detection, the participation of the silica (SiO_2_) filler in the overall co-pyrolysis chemistry cannot be fully excluded. Partially fluorinated hydrocarbons and hydrogen-terminated fluorocarbons are formed in comparable amounts, based on the semi-quantitative analysis, irrespective of the applied mixture ratio. Fluorinated silanes are present only in minor relative abundances within the detected product spectrum. Figure 3 presents the relative abundance of the gaseous products, categorized by origin and product group. The most abundant compounds within each category, identified by library matching, are listed in Table 3, whereas a comprehensive overview of all additionally identified compounds is provided in Table S2. Notably, all fluorine-containing compounds identified in the gaseous phase, apart from the fluorinated silanes, fall within the OECD definition of PFAS. Although the virgin polymer mixtures and the composites contain largely the same set of identified compounds, their relative abundance follows a consistent overall trend that is more distinctly expressed in the composite materials. Several individual species, however, deviate from this pattern. These discrepancies are likely linked to differences in polymer structure, particularly the degree of crosslinking in the SR and its consequent influence on the degradation pathways.

Regarding the potential for recycling, the concurrent formation of TFE together with a diverse set of fluorinated and partially fluorinated byproducts could be particularly problematic. Although TFE is a valuable monomer, its coexistence with numerous fluorinated compounds that fall within the OECD definition of PFAS could significantly complicate downstream separation and purification. This complex mixture of PFAS could further complicate product handling, purification, and the selective recovery of valuable products. Consequently, the recovery of polymer-grade TFE from co-pyrolysis gas streams could require more demanding separation and purification processes than recovery from pure PTFE pyrolysis, potentially limiting monomer recovery under mixed-polymer conditions.

### 3.5. Analysis of Liquid and Solid Products

In addition to the liquid phase, co-pyrolysis of SR and PTFE, as well as pyrolysis of the corresponding composite materials, yields small amounts of a white solid, analogous to that obtained during pure PTFE pyrolysis. The FTIR spectra (Figure S2) of these isolated solids closely matched those of virgin PTFE-V, suggesting that oligomeric PTFE is still formed to a small extent during co-pyrolysis with SR. The vibration bands of 1202 cm^−1^ and 1146 cm^−1^ can be attributed to the asymmetric and symmetric CF_2_ stretching, while the peak at 639 cm^−1^ corresponds to CF_2_ wagging vibrations, which is in accordance with the literature [47,48,49]. The recombination of difluorocarbene during the pyrolysis of pure PTFE at cold spots, yielding oligomeric PTFE, has been reported in the literature [19].

The pyrolysis of SR in the presence of PTFE results in a chemically complex mixture comprising both siloxane and fluorinated species. SR-derived pyrolysis products were identified as cyclic siloxanes (D_x_) with different ring sizes by comparison with the literature data [50], reference substances and the NIST database. However, other product classes formed during co-pyrolysis are mostly not included in commercially available databases and neither reference materials nor published data are available for comparison. As a result, the assignment of these compounds relies heavily on manual interpretation of mass-spectral fragmentation patterns and comparison with known fragmentation patterns of compounds found in databases and the literature.

Overall, the co-pyrolysis products can be classified into different product groups depending on their fragmentation pattern and chemical structure. The assigned product groups are cyclic siloxanes (D_x_), mono- (D_x_F) and difluorinated cyclic siloxanes (D_x_F_2_), difluorinated linear siloxanes (L_x_F_2_), 1H-fluorocarbons (C_x_F_2x+1_), unassigned fraction (U_x_) and minor amounts of different siloxane compounds with no clear structural motif. Notably, the assignment of D_x_F, D_x_F_2_ and L_x_F_2_ was conducted through interpretation of MS data and comparison to the non-fluorinated counterparts (D_x_). C_x_F_2x+1_ compounds were identified from specific reference spectra (e.g., C_6_F_13_H) if available, from the Wiley database. If no reference spectrum was available, the mass spectrum was manually interpreted by comparison with homologous or structurally analogous compounds, allowing systematic differences in molecular mass and fragmentation behavior to be evaluated. Therefore, the assignment of these structures should be considered tentative.

**Figure 4:**
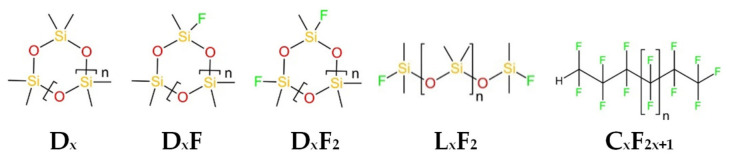
Proposed major liquid product groups formed during PTFE:SR co-pyrolysis based on GC–MS and NMR analysis. Structural assignments are tentative and were derived primarily from mass spectral interpretation.

Figure 4 summarizes the main product categories formed during pyrolysis. A detailed list of all liquid products and the rationale of their assignment can be found in Table S3 of the Supplementary Material. Among the pyrolysis products of the pure polymers, the D_x_ fraction represents the only product group derived from pure SR degradation. Their presence in the liquid fraction obtained from the co-pyrolysis indicates that the original degradation mechanism of SR decomposition is not completely suppressed under these conditions. All other compound classes can be attributed to co-pyrolytic interactions between SR and PTFE. The same product groups were formed for the PTFE-V:SR-V, PTFE-T:SR-T mixtures and composite materials. Among the identified product fractions, only the C_x_F_2x+1_ compounds fall within the OECD definition of PFAS.

### 3.6. Liquid Product Identification

The assigned fluorinated siloxanes (D_x_F, D_x_F_2_, L_x_F_2_) exhibit mass spectral characteristics like their non-fluorinated analogs. Molecular ions generally appear at very low intensities, which is the typical fragmentation pattern reported for siloxanes. At high *m*/*z* values the observed spectra are consistently shifted by +4 *m*/*z* for monofluorinated (D_x_F) and +8 *m*/*z* for difluorinated siloxanes (D_x_F_2_, L_x_F_2_). This behavior is consistent with the substitution of one and two methyl groups (CH_3_, 15 Da) by one and two fluorine atoms (F, 19 Da), respectively. Notably, no fragment ions indicative of Si–F bond cleavage (Δ*m*/*z* = 19) are observed, suggesting that dissociation of this bond is an uncommon fragmentation pathway under the applied ionization conditions, which could be attributed to the high Si-F bond energy [20]. At lower *m*/*z* values, the fragmentation pattern closely matches that of non-fluorinated siloxanes. This resemblance possibly arises from cleavage of the fluorine-containing substituent, followed by ionization of the remaining non-fluorinated backbone. However, double peaks shifted by Δ*m*/*z* = 4 or Δ*m*/*z* = 8 remain detectable, although only with low intensities. For the potential difluorinated siloxanes the observed ions imply that the simultaneous substitution of two methyl groups on a different silicon atom is more favored. Difluorinated species primarily display fragments shifted by only +4 *m*/*z* after loss of a single fluorinated moiety (e.g., Δ*m*/*z* = 78, SiOFCH_3_ fragment), indicating that the second fluorine substituent is on a different silicon atom.

For the unassigned fraction (U_x_), no unambiguous chemical structure could be assigned based on the available analytical data. GC-MS analysis revealed a fragmentation pattern closely resembling that of the monofluorinated and difluorinated cyclic siloxanes (D_x_F, D_x_F_2_), with the notable exception of an additional fragment at +44 *m*/*z* relative to the corresponding D_x_F or D_x_F_2_ base peak. This similarity suggests that the U_x_ fraction could be structurally related to the fluorinated siloxane species identified in this study. However, the available analytical data does not allow reliable structural elucidation or differentiation between possible structural motifs. Consequently, the exact chemical structure of the U_x_ fraction remains unresolved and would require additional analytical techniques.

**Figure 5:**
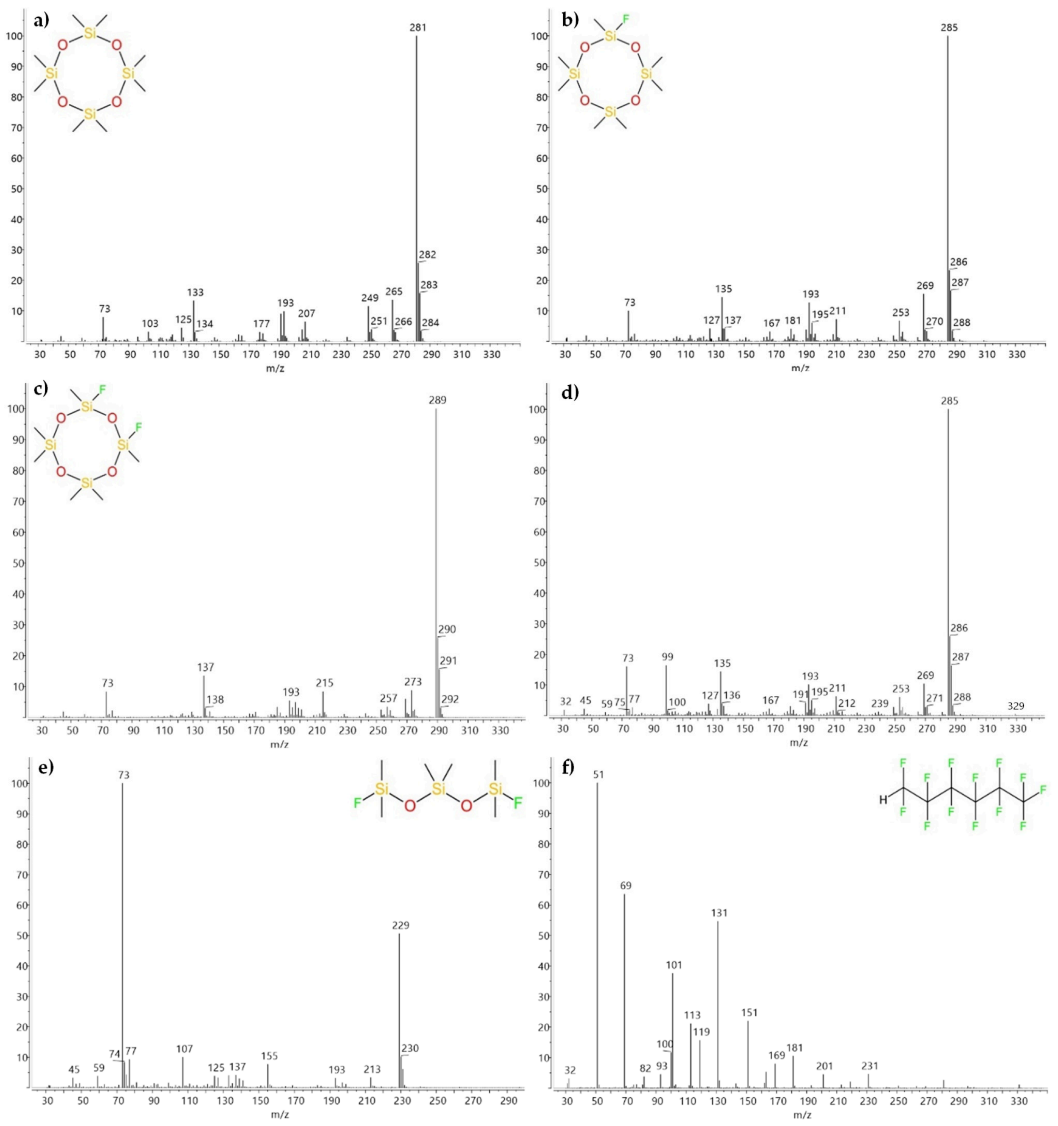
Representative mass spectra of the major liquid product groups identified during PTFE:SR co-pyrolysis: (**a**) D_x_, (**b**) D_x_F, (**c**) D_x_F_2_, (**d**) U_x_, (**e**) L_x_F_2_, and (**f**) C_x_F_2x+1_, together with their proposed structures. Relative to Dx, the characteristic fragment ions of D_x_F and D_x_F_2_ are shifted by +4 *m*/*z* and +8 *m*/*z*, respectively.

The C_x_F_2x+1_ product group in both the liquid and gaseous products exhibits notable deviations from the characteristic fragmentation behavior typically observed in fully perfluorinated compounds. Rather than displaying a base peak at *m*/*z* = 69 (CF_3_^+^), the mass spectra are dominated by a signal at *m*/*z* = 51 (CF_2_H^+^), indicating the presence of hydrogen in the terminal groups. In addition, the dominant neutral losses within this class, Δ*m*/*z* = 20, could correspond to HF elimination, and Δ*m*/*z* = 50, corresponding to the loss of a CF_2_ group, further supports the presence of partially hydrogenated fluorocarbon structures. The simultaneous presence of peaks at *m*/*z* = 69 and *m*/*z* = 51 suggests that members of the C_x_F_2x+1_ group are terminated by -CF_3_ and -CF_2_H groups. The increase of +50 *m*/*z* (=CF_2_) between the different peaks indicates longer chains. Interestingly, such species are absent from pyrolysis products of pure PTFE, where difluorocarbene recombination can lead to the formation of oligomeric PTFE fragments in cold spots during pyrolysis, a behavior consistently reported in the literature [11] and confirmed in the present experiments. During co-pyrolysis, this recombination pathway may be partially suppressed because hydrogen abstraction from the co-pyrolyzed polymer matrix can divert the degradation chemistry. As a result, short chain 1H-fluorocarbons in liquid (C_5_F_11_H–C_8_F_17_H) and gaseous products could be formed alongside higher fluorinated oligomers as the isolated white solids. Figure 5 highlights the MS fragmentation pattern of each main product group using one representative example.

The ^29^Si-NMR analysis shows that cyclic siloxane species constitute the major products, with resonances appearing between −8 and −23 ppm. This chemical-shift range is characteristic of internal siloxane units in cyclic or long chained linear siloxane structures. Upon introducing PTFE into the feed material, the spectrum becomes significantly more complex, exhibiting numerous additional resonances spanning from −3.5 to −23.0 ppm with the majority of resonances occurring between −15.0 and −23.0 ppm. This suggests the formation of a broader variety of linear siloxanes, caused by interactions or side reactions between PTFE degradation products and the siloxane matrix. Additionally, a noticeable decrease in the intensity of peaks associated with D_x_ units suggests partial replacement or modification of these environments. The formation of Si-F bonds should result in a more upfield chemical shift due to the strong electronegativity of fluorine, which aligns with the observed spectral changes. The formation of trifunctional (RSiO_3_) and tetrafunctional (SiO_4_) units is unlikely, as these species typically resonate further upfield (<−55 ppm), where no signals were observed [14]. 

The ^19^F-NMR exhibits two regions containing a multitude of peaks, −60 to −70 ppm and −120 to −140 ppm. The region of −120 to −140 ppm may correspond to the different fluorosiloxanes as well as the -CF_2_- of the C_x_F_2x+1_ group. The region from −60 to −70 ppm possibly corresponds to -CF_3_ groups from the C_x_F_2x+1_ group [51,52]. Notably, peaks corresponding to HF [53] could not be detected in the liquid products, suggesting that HF was not present in detectable amounts in the analyzed liquid fraction. However, HF was not directly monitored during pyrolysis. If formed, HF may have remained predominantly in the gas phase, reacted with moisture, reactor surfaces, or other pyrolysis products before analysis, or been present below the detection limit of the applied methods. Overall, the NMR data is in good agreement with the results from GC-MS analysis. The NMR spectra are shown in Figures S3 and S4.

### 3.7. Liquid Product Composition

**Figure 6:**
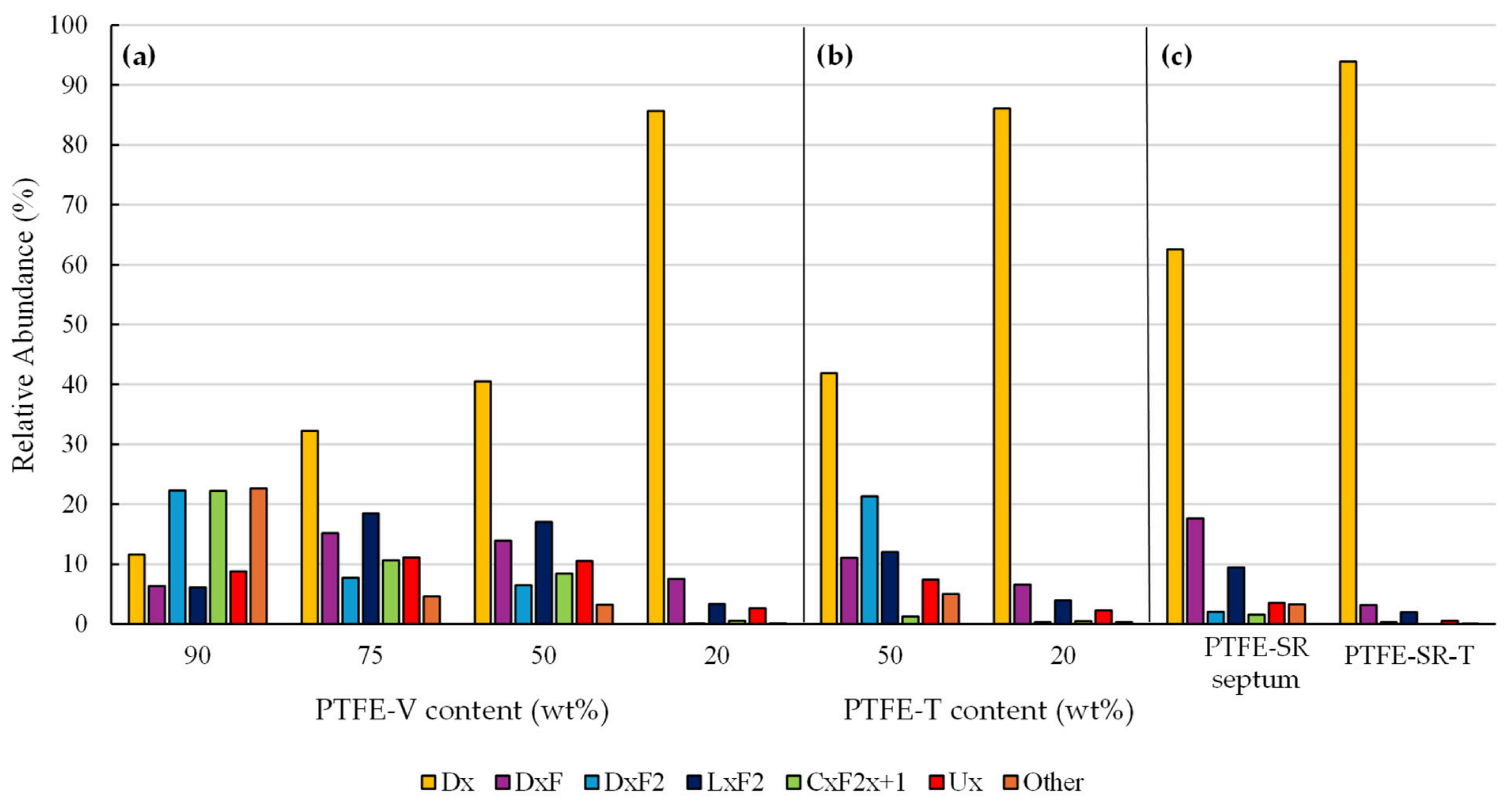
Relative abundance determined by GC–MS for (**a**) PTFE-V:SR-V mixtures, (**b**) PTFE-T:SR-T mixtures, and (**c**) composite materials.

The distribution of the individual product groups for PTFE:SR mixtures was determined semi-quantitively via peak area normalization and exhibited a non-linear dependence on the PTFE content, as shown in Figure 6a. A substantial shift in the product distribution occurs between 20 wt% and 50 wt% PTFE, where the relative abundance in all liquid fluorinated components (C_x_F_2x+1_, D_x_F, D_x_F_2_, L_x_F_2_, U_x_) increase markedly, while the relative abundance of D_x_ decreases. In contrast, increasing the PTFE content from 50 wt% to 75 wt% results only in a minor increase in the relative abundance in liquid fluorinated products, indicating that their formation becomes progressively less sensitive to additional PTFE at high loadings. For mixtures containing high PTFE fractions (90 wt%), the relative proportions deviate substantially from these trends, most likely because the greatly increased concentration of PTFE-derived intermediates promotes competing side reactions. The pronounced changes observed at intermediate PTFE contents suggest that polymer interactions become increasingly important once sufficient fluorinated degradation products are generated to compete with the intrinsic degradation pathways of SR. At low PTFE contents, the concentration of reactive fluorinated intermediates is likely insufficient to substantially modify the siloxane degradation chemistry. As the PTFE fraction increases, however, the probability of interactions between fluorinated species and silicone-derived degradation intermediates increases, promoting competitive reaction pathways such as fluorination, hydrogen abstraction, and C–Si bond cleavage. These competing reactions could alter the siloxane degradation pathways, thereby decreasing the relative abundance of cyclic siloxanes while increasing the formation of fluorinated products. Although the exact elementary reaction mechanisms remain unresolved, the observed non-linear behavior indicates that the extent of polymer interaction depends not only on the presence of PTFE but also on the relative abundance of reactive degradation intermediates generated during co-pyrolysis. In addition, increasing PTFE content increases the interfacial contact between both polymer phases, thereby potentially enhancing the probability that fluorinated degradation intermediates encounter silicone-derived species before leaving the reaction zone. Consequently, both the abundance of reactive fluorinated intermediates and the degree of polymer contact are expected to contribute to the observed transition in product distribution. The relative abundance of D_x_ decreases sharply with rising PTFE content, which is likely attributable to enhanced competing side reactions that could suppress D_x_ formation during silicone degradation. Conversely, the relative abundance of C_x_F_2x+1_ increases with increasing PTFE content, presumably due to the higher availability of CF_2_-containing radicals in the gas phase, which could facilitate recombination leading to perfluorinated fragments. The identified 1H-fluorocarbons (C_x_F_2x+1_) detected in the liquid products fall within the OECD definition of PFAS [32]. Their structural assignment is based on GC–MS fragmentation patterns supported by ^19^F-NMR analysis and should therefore be regarded as tentative where authentic reference spectra were unavailable. Although PTFE decomposition produces PFAS in the gaseous and solid phase, the presence of this compound class in the liquid pyrolysis products could introduce additional challenges for recycling, separation and environmental management. The L_x_F_2_-type fluorinated siloxanes retain a siloxane backbone, suggesting that they could, in principle, serve as potential precursors for repolymerization into modified silicone materials. This possibility is supported by prior demonstrations of repolymerization for L_2_F_2_ and SiMe_2_F_2_ [54]. However, their effective reuse would most likely require selective isolation from the complex product mixture. By contrast, the partially fluorinated cyclic siloxanes D_x_F and D_x_F_2_ have, to date, not found any significant industrial application. These results demonstrate that PTFE concentration not only affects product distribution but also governs the overall product selectivity of the degradation process. The pronounced increase in newly formed compounds accounts for the sharp rise in liquid-phase yield observed during co-pyrolysis and the decrease in gaseous yield. Importantly, the overall product distribution and the principal product classes obtained from PTFE-V:SR-V mixtures closely resemble those observed for the investigated PTFE-T:SR-T materials under the applied experimental conditions (Figure 6b). This suggests that the virgin-polymer mixtures capture the dominant co-pyrolysis behavior of these specific materials. However, because the exact formulations of the commercial materials are not fully known, this comparison should be regarded as qualitative and should not be generalized to all silicone/PTFE composite waste systems. Importantly, the strong dependence of the product spectrum on PTFE content implies that composites (Figure 6c) containing only small amounts of PTFE produce a high relative abundance of valuable cyclic siloxanes (D_x_). Additionally, C_x_F_2x+1_-type PFAS are below the limit of detection under the applied analytical conditions (corresponding to a relative abundance below 0.01%). Such composites retain substantially higher relative abundance of D_x_ while limiting the formation of fluorinated products under the investigated conditions. These characteristics suggest that low-PTFE composites may represent more favorable feedstocks for depolymerization-based recycling than PTFE-rich systems. However, practical recycling feasibility would additionally depend on product purity, separation efficiency, contaminant tolerance and process design. This behavior is exemplified by the investigated PTFE-SR-T composite, where the low PTFE fraction results in a product distribution characterized by a lower relative abundance of fluorinated products and a higher relative abundance of D_x_ species under the investigated conditions. The product distribution of different pyrolysis experiments is summarized in Table S4.

**Figure 7:**
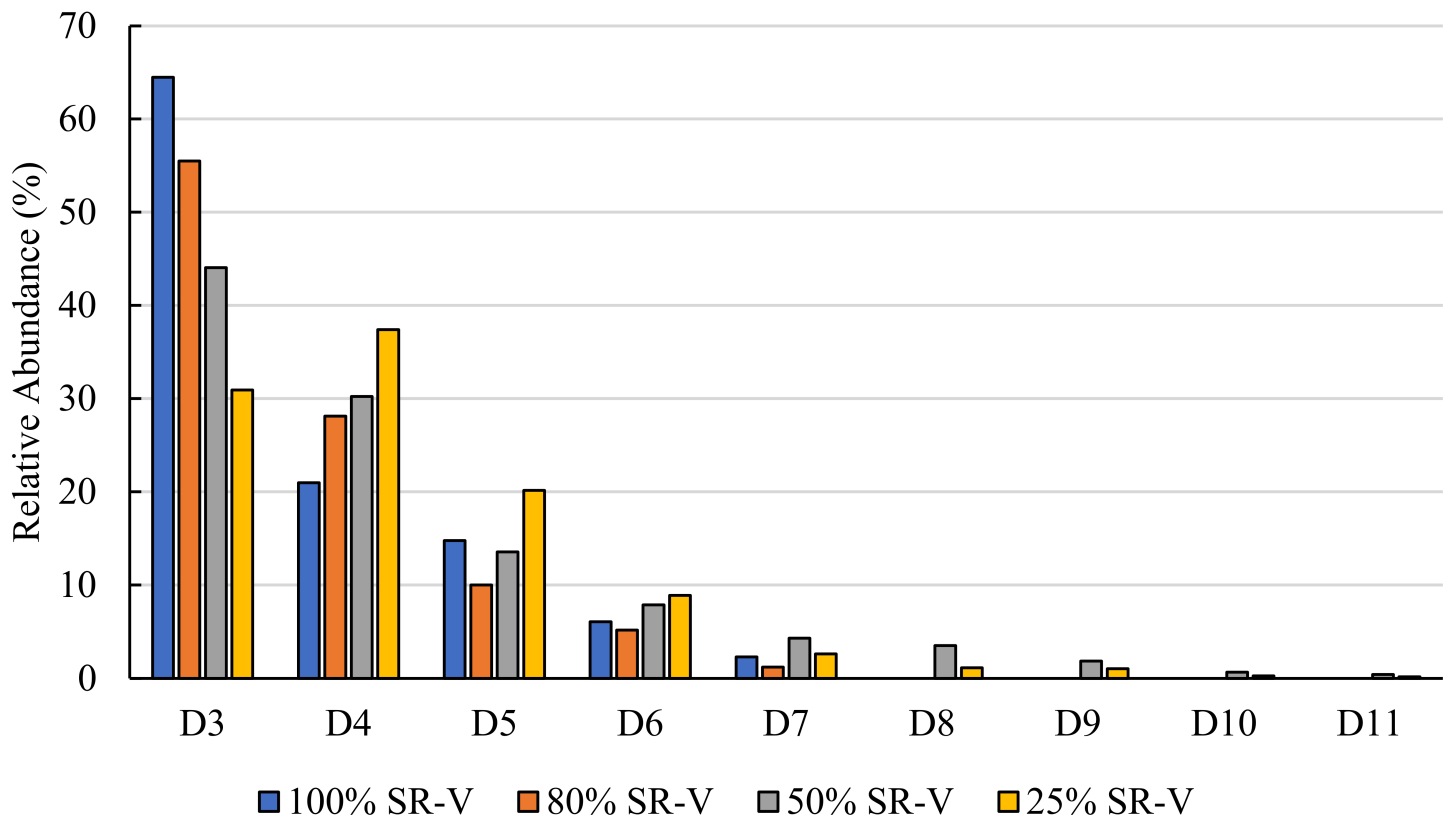
Relative abundance of cyclic siloxanes (D_x_) in the liquid products obtained from PTFE-V:SR-V mixtures at different mixing ratios.

Overall, co-pyrolysis with PTFE promotes the formation of larger cyclic siloxanes, with species up to D_11_ detected at low relative abundances (0.1%). However, no clear trend is observed for siloxanes larger than D_8_. While the formation of higher cyclic siloxanes is well documented in the literature [14], these compounds were below the limit of detection in samples without PTFE. This suggests that the presence of PTFE may facilitate or enhance the formation of these larger cyclic siloxanes. The cyclic siloxane (D_x_) distribution for PTFE-V:SR-V mixtures is shown in Figure 7. Notably, the chain length distribution of the proposed L_x_F_2_ product fraction closely resembles that of the D_x_ species. The L_x_F_2_ compounds exhibit chain lengths comparable to the ring sizes of D_x_ oligomers (up to 13 SiMe_2_O units), whereas cyclic fluorinated siloxanes are detected only up to a ring size of six siloxane units. 

### 3.8. Mechanistic Insights

The persistence of the characteristic degradation products from the individual polymers, namely cyclic siloxanes from SR and TFE and OFCB from PTFE, demonstrates that the primary pyrolysis pathways of both materials remain active during co-pyrolysis. At the same time, the formation of entirely new product groups indicates that the co-pyrolysis chemistry cannot be explained by a simple additive combination of the individual degradation mechanisms.

The formation of partially hydrogenated fluorocarbons in both the gaseous and liquid fractions suggests that CF_2_-derived radicals generated during PTFE degradation undergo recombination reactions while also participating in hydrogen abstraction from the siloxane matrix. Such hydrogen abstraction reactions could compete with the typical recombination pathways of difluorocarbene, resulting in the formation of the C_x_F_2x+1_ product group. Likewise, the detection of fluorinated siloxanes (D_x_F and D_x_F_2_) is consistent with fluorine transfer or substitution reactions between PTFE-derived intermediates and SR degradation products, leading to replacement of methyl substituents at silicon by fluorine.

The simultaneous detection of linear difluorinated siloxanes (L_x_F_2_) and difluorodimethylsilane is consistent with Si–O bond cleavage during co-pyrolysis, although the exact reaction pathway cannot be determined from the present data. Despite the high bond dissociation energy of the Si–O bond (452 kJ mol^−1^) [55], highly reactive fluorocarbon radicals may facilitate bond cleavage, promoting ring opening and the formation of linear siloxane structures. The reduced residue formation observed during co-pyrolysis could be attributed to partial suppression of silicone cross-linking reactions. This may contribute to the lower residue yield, while concurrent reactions involving PTFE-derived intermediates could reduce the gaseous product fraction by converting volatile fluorinated species into higher molecular weight products. The resulting incorporation of fluorine, which forms one of the strongest bonds to silicon (565 kJ mol^−1^) [55], may explain the prevalence of linear fluorinated siloxanes in the product mixture. The product groups of fluorinated cyclic siloxanes (D_x_F and D_x_F_2_) are consistent with the substitution of a methyl group with fluorine. The fluorination of PDMS-based silicone rubber has been reported in the literature, using F_2_ gas [56] or CF_4_ plasma [57,58]. Although these studies support the substitution of CH_2_- to CH_2_F-groups, there is currently insufficient evidence to confirm analogous substitution processes under pyrolytic conditions. This limitation also applies when considering the unidentified Ux fraction. Nevertheless, the observed products suggest that PTFE-derived degradation intermediates may exhibit fluorinating behavior under the investigated conditions. Si–O bond cleavage by fluorinated species has previously been demonstrated for SiO_2_ during reactive-ion and plasma etching using CF_4_ and CF_4_/O_2_ atmospheres [59]. While these observations support the plausibility of fluorine-induced Si–O bond activation, they should not be regarded as direct evidence that identical processes occur during PTFE:SR co-pyrolysis, as such reactions have not been demonstrated for silicone materials under pyrolytic conditions. Overall, the observed product distributions are consistent with a mechanistic framework involving hydrogen abstraction, fluorine substitution, and Si–O bond cleavage. While the proposed pathways are inferred from product analysis, they provide a valuable basis for future experimental and computational studies directed at elucidating the detailed reaction mechanisms governing PTFE–SR co-pyrolysis.

## 4. Conclusions

This study demonstrates that the co-pyrolysis of silicone rubber (SR) and polytetrafluoroethylene (PTFE) under inert laboratory-scale conditions leads to depolymerization pathways and product distributions that cannot be described by a simple superposition of the respective single-polymer behaviors. While both SR and PTFE can be efficiently depolymerized into monomers or low-molecular-weight oligomers during pyrolysis of the pure polymers, their combination results in pronounced cross-polymer interactions. Mass-balance analysis reveals an increase in the liquid product fraction of up to 30 wt% during co-pyrolysis, accompanied by a decrease in both gaseous products and solid residue compared to values calculated from individual polymer pyrolysis. These deviations could be attributed to the suppression of silicone cross-linking reactions, which reduces residue formation, and to the consumption of PTFE-derived volatile intermediates in cross-polymer reactions, which limits gas-phase product formation. Importantly, the same principal product fractions were observed for virgin polymer mixtures, commercial tubing materials, and commercial composite products. Although differences in relative abundances were detected, the overall product spectrum remained largely unchanged, indicating that the fundamental co-pyrolysis reactions are similar across all investigated materials. While these findings should not be directly generalized to all PTFE-containing silicone composites, they provide a promising basis for future investigations into the influence of formulation, additives, filler content and crosslink density on co-pyrolysis behavior. Comprehensive product identification by GC–MS, supported by NMR and FTIR analyses, shows that the enriched liquid phase contains not only the expected cyclic siloxanes (D_x_), but also newly formed product classes absent in single-polymer experiments. These compounds were assigned tentatively through MS spectral interpretation. They include fluorinated siloxanes (D_x_F, D_x_F_2_, L_x_F_2_) and per- and polyfluoroalkyl substances (C_x_F_2x+1_). Additional partially fluorinated hydrocarbons and fluorinated silanes were identified in the gaseous phase via library matches.

Among the fluorinated siloxane products, L_x_F_2_ species retain a siloxane backbone and therefore remain structurally related to conventional silicone intermediates, suggesting potential relevance for repolymerization. However, effective utilization would require selective isolation from chemically complex mixtures, whereas partially fluorinated cyclic siloxanes (D_x_F, D_x_F_2_) currently lack established industrial applications. Despite these challenges, the yield of cyclic siloxanes (D_x_), which are the target products for silicone depolymerization processes, remains comparatively high at low PTFE contents. Regarding the potential for recycling, the concurrent formation of TFE together with a diverse mixture of substances classified as PFAS increases the complexity of the gaseous product stream compared to the pyrolysis of pure PTFE. Furthermore, the co-pyrolysis of PTFE and SR results in the formation of the C_x_F_2x+1_ product group, which falls within the OECD definition of PFAS and, unlike the primary degradation products of pure PTFE that are predominantly detected in the gaseous or solid fractions, is also present in the liquid pyrolysis product. This broader distribution of PFAS across multiple product streams could complicate downstream product handling and purification. Consequently, recovery of polymer-grade TFE from such mixed product streams likely requires more demanding separation and purification processes than recovery from pure PTFE pyrolysis. Although the observed product distribution provides strong evidence for interactions between PTFE- and silicone-derived degradation products, the proposed reaction pathways should be regarded as mechanistic interpretations based on indirect analytical evidence rather than direct observation of the underlying elementary reactions. Furthermore, while the present study identified only compounds classified as PFAS according to the OECD definition, the results provide an important foundation for future investigations into their environmental relevance, including assessments of toxicity, environmental fate, and removal efficiencies. Such studies will further improve the understanding of fluorinated products formed during co-pyrolysis and support the development of effective management and recycling strategies in light of evolving regulatory requirements.

## Figures and Tables

**Table 1:** TGA parameters for SR-V, PTFE-V, mixtures and PTFE:SR composites. T_P_ = peak degradation temperature; T_O_ and T_E_ = temperatures corresponding to 1% of the maximum weight-loss rate before and after T_P_, respectively; ΔT = T_E_ − T_O_.

	T_O_	T_P_	T_E_	ΔT	Residue
	°C	°C	°C	°C	(wt%)
SR-V	308.1	588.8	723.3	415.2	34.82
PTFE-V	509.4	597.4	610.4	101.0	0.08
PTFE-SR septum	421.2	522.6	580.5	159.3	40.65
PTFE-SR-T	274.6	682.9	756.5	481.9	30.95
PTFE-V:SR-V 20:80	443.4	616.7	649.3	205.9	16.10
PTFE-V:SR-V 50:50	490.6	626.2	644.0	153.3	8.02
PTFE-V:SR-V 75:25	416.8	638.5	650.7	233.9	6.33

**Table 2:** Mass balancing of PTFE, SR and composite pyrolysis.

	Gaseous	Liquid	Residue
	wt%	wt%	wt%
PTFE-V	96.90 ± 0.03	2.87 ± 0.08	0.22 ± 0.06
SR-V	3.56 ± 0.08	47.65 ± 0.13	48.78 ± 0.20
SR-T	2.82 ± 0.13	53.02 ± 0.13	44.16 ± 0.05
PTFE-T	94.72 ± 0.19	4.87 ± 0.18	0.42 ± 0.03
PTFE-SR septum	19.47 ± 0.13	35.62 ± 0.14	44.90 ± 0.21
PTFE-SR-T	10.01 ± 0.18	50.88 ± 0.13	39.10 ± 0.08

**Table 3:** Major gaseous degradation products identified from PTFE:SR co-pyrolysis and their corresponding normalized peak areas obtained from the different composite materials and polymer mixtures. The peak areas were determined semi-quantitatively to compare relative changes under identical conditions.

		Relative GC-MS Peak Area (Normalized to 100%) (%)				
**Main SR derived products**		**PTFE-V**	**PTFE-V:SR-V 3:1**	**PTFE-V:SR-V 1:1**	**PTFE-SR septum**	**PTFE-SR-T**
Methane	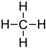	-	0.1	1.1	8.2	6.4
Ethane	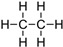	-	1.0	1.5	1.2	1.3
**Main PTFE derived products**		**PTFE-V**	**PTFE-V:SR-V 3:1**	**PTFE-V:SR-V 1:1**	**PTFE-SR septum**	**PTFE-SR-T**
Tetrafluoroethylene	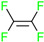	18.4	15.7	13.7	14.7	11.7
Octafluorocyclobutane	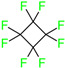	29.6	22.8	18.3	7.8	8.8
Hexafluoropropene	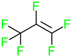	36.4	24.1	19.1	6.6	9.8
**Main partially fluorinated hydrocarbons**		**PTFE-V**	**PTFE-V:SR-V 3:1**	**PTFE-V:SR-V 1:1**	**PTFE-SR septum**	**PTFE-SR-T**
1,1-Difluoroethene	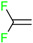	-	1.4	3.0	5.4	5.4
1,1,1,2-Tetrafluoroethane	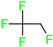	-	0.1	0.5	2.8	2.0
3,3,3-Trifluoropropene	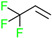	-	8.5	10.8	2.6	2.2
**Main H-terminated fluorocarbons**		**PTFE-V**	**PTFE-V:SR-V 3:1**	**PTFE-V:SR-V 1:1**	**PTFE-SR septum**	**PTFE-SR-T**
Trifluoroethene	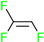	-	1.0	2.0	3.15	2.5
Difluoromethane	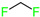	-	0.3	1.1	5.2	4.9
1,1,2,2-Tetrafluoroethane	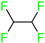	-	0.9	1.7	6.1	4.9
Pentafluoroethane	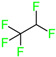	-	4.1	5.3	5.4	5.8
**Main fluorinated silanes**		**PTFE-V**	**PTFE-V:SR-V 3:1**	**PTFE-V:SR-V 1:1**	**PTFE-SR septum**	**PTFE-SR-T**
Trimethylfluorosilane	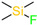	-	3.0	2.9	5.4	2.6

## Data Availability

The original contributions presented in this study are included in the article/Supplementary Materials. Further inquiries can be directed to the corresponding author.

## Supplementary Materials

The following supporting information can be downloaded at: https://www.mdpi.com/article/10.3390/polym18151929/s1, Figure S1: TGA of PTFE-SR septum with a temperature hold of 30 min at 400 °C; Figure S2: FTIR spectra of PTFE-V and of the white solid obtained during PTFE-V and PTFE-V:SR-V mixture pyrolysis; Figure S3: ^29^Si-NMR of PTFE-V:SR-V mixture (1:1) liquid product; Figure S4: ^19^F-NMR of PTFE-V:SR-V mixture (1:1) liquid product; Table S1: Individual mass-balance results of two independent experimental runs for the investigated polymer mixtures; Table S2: Compounds identified in the gaseous products of PTFE-V, PTFE-V:SR-V and composites; Table S3: Fragmentation pattern, normalized peak area (100%) and possible structure of PTFE-V:SR-V liquid products at varying PTFE-V content determined via GC-MS; Table S4: Relative abundance of different product groups determined by normalized GC–MS peak areas of different pyrolysis experiments for PTFE-V:SR-V mixtures and composite materials.

## Abbreviations

The following abbreviations are used in this manuscript:

C_2_H_x_	Summary for C_2_-hydrocarbons, namely ethane and ethene
D_x_	Cyclic siloxanes (x = number of siloxane units)
D_x_F	Monofluorinated cyclic siloxanes
D_x_F_2_	Difluorinated cyclic siloxanes
FTIR	Fourier-transform infrared spectroscopy
GC	Gas chromatography
HFP	Hexafluoropropene
L_x_F_2_	Difluorinated linear siloxanes
*m*/*z*	Mass-to-charge ratio
NIST	National Institute of Standards and Technology
NMR	Nuclear magnetic resonance
OECD	Organisation for Economic Co-operation and Development
OFCB	Octafluorocyclobutane
PDMS	Polydimethylsiloxane
PFAS	Per- and polyfluoroalkyl substances
PTFE	Polytetrafluoroethylene
PTFE-SR-T	PTFE-lined silicone rubber tubing
PTFE-T	Polytetrafluoroethylene tubing
PTFE-V	Virgin polytetrafluoroethylene
SR	Silicone rubber
SR-T	Silicone rubber tubing
SR-V	Virgin silicone rubber
T_o_	Onset temperature for TGA
T_E_	End temperature for TGA
T_P_	Peak degradation temperature for TGA
TFE	Tetrafluoroethylene
TGA	Thermogravimetric analysis
U_x_	Unassigned fraction
$x_{l,g,r}^{calc}$	Calculated mass fraction of product (liquid, gaseous, residue) of a specific polymer mixture
$x_{l,g,r}^{i}$	Mass fraction of product (liquid, gaseous, residue) derived from the pyrolysis of the pure polymer i
m_i_	Mass fraction of polymer i in starting material
ΔT	Degradation interval for TGA
