# Supplementary material for: Thermal Depolymerization Challenges of PTFE:Silicone Rubber Mixtures and Composite Materials
Source: Polymers (Basel). 2026 Aug 6;18(15):1929. doi: 10.3390/polym18151929 (PMC13468768; doi:10.3390/polym18151929)
Supplement: Supplementary file 1 [file polymers-18-01929-s001.zip › polymers-4437880-supplementary.pdf]

Supplementary Material

for

Thermal Depolymerization Challenges of PTFE:Silicone Rubber Mixtures and Composite Materials

Lukas Eigenschink[1,2*], Matthias Mastalir[3], Michael Harasek[2], Christian Paulik[1]

[1] Institute for Chemical Technology of Organic Materials, Johannes Kepler University Linz, Altenberger Straße 69, 4040 Linz, Austria; christian.paulik@jku.at
[2] Institute of Chemical, Environmental and Bioscience Engineering, TU Wien, Getreidemarkt 9/166, 1060 Vienna, Austria; michael.harasek@tuwien.ac.at
[3] OMV Downstream GmbH, Mannswörther Straße 28, 2320 Schwechat, Austria; matthias.mastalir@omv.com
* Correspondence: lukas.eigenschink@jku.at

Figure S1: TGA of PTFE-SR septum with a temperature hold of 30 min at 400°C 2
Figure S2: FTIR spectra of PTFE-V and of the white solid obtained during PTFE-V and PTFE-V:SR-V mixture pyrolysis 3
Figure S3: ^{29}Si-NMR of PTFE-V:SR-V mixture (1:1) liquid product 4
Figure S4: ^{19}F-NMR of PTFE-V:SR-V mixture (1:1) liquid product 4

Table S1: Individual mass-balance results of two independent experimental runs for the investigated polymer mixtures.. 5
Table S2: Compounds identified in the gaseous products of PTFE-V, PTFE-V:SR-V and composites 5
Table S3: Fragmentation pattern, normalized peak area (100%) and possible structure of PTFE-V:SR-V liquid products at varying PTFE-V content determined via GC-MS. 8
Table S4: Relative abundance of different product groups determined by normalized GC–MS peak areas of different pyrolysis experiments for PTFE-V:SR-V mixtures and composite materials 12

* Corresponding author
E-Mail address: lukas.eigenschink@jku.at

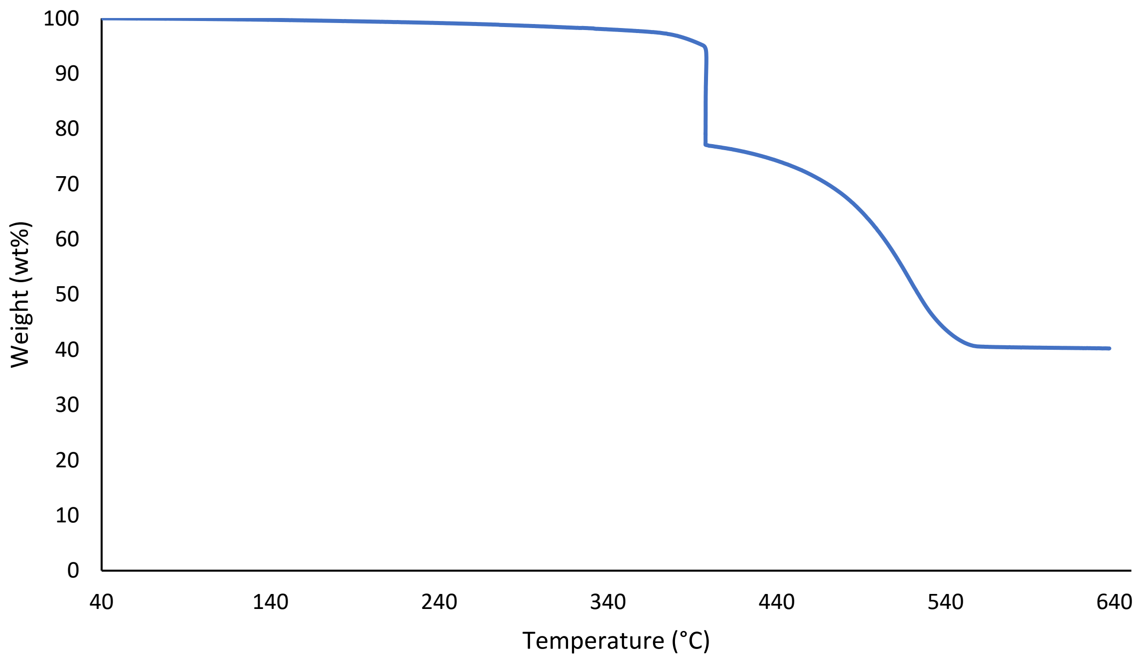

Figure S1: TGA of PTFE-SR septum with a temperature hold of 30 min at 400°C

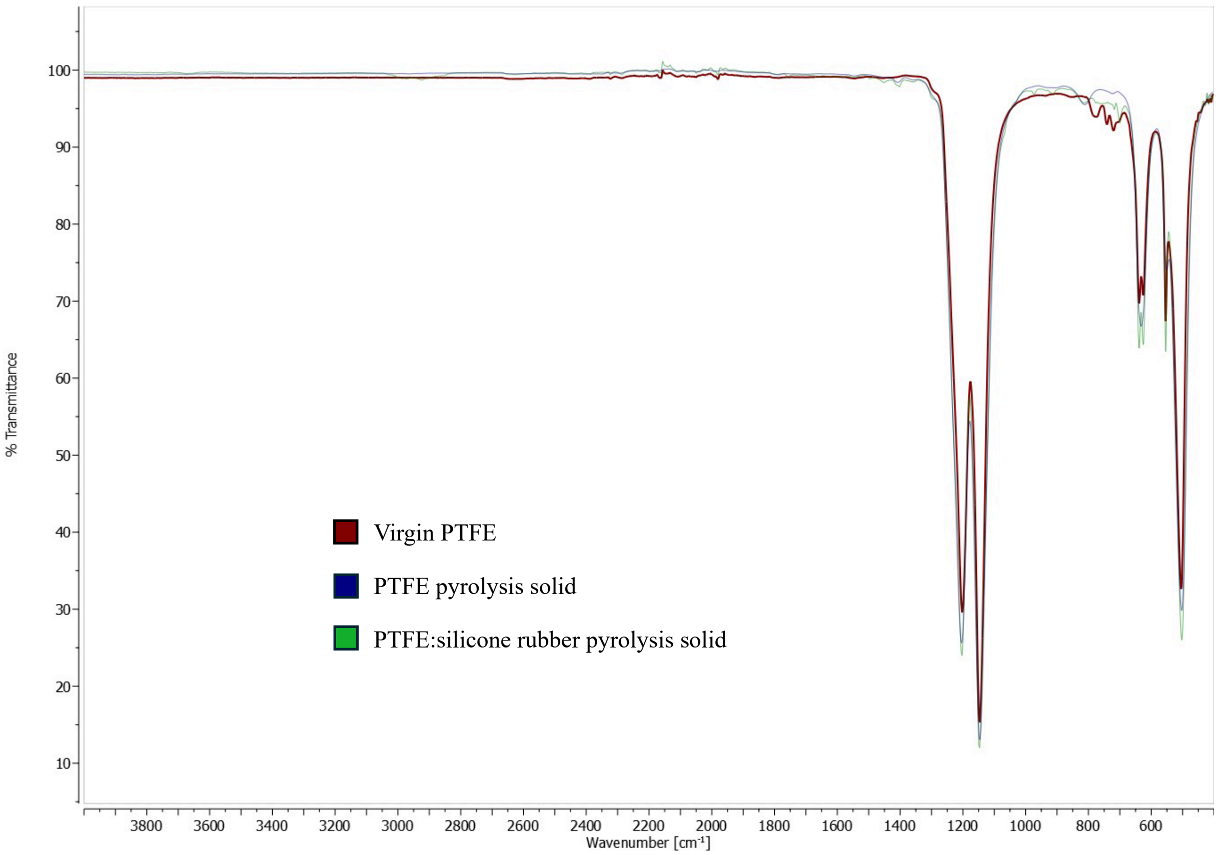

Figure S2: FTIR spectra of PTFE-V and of the white solid obtained during PTFE-V and PTFE-V:SR-V mixture pyrolysis

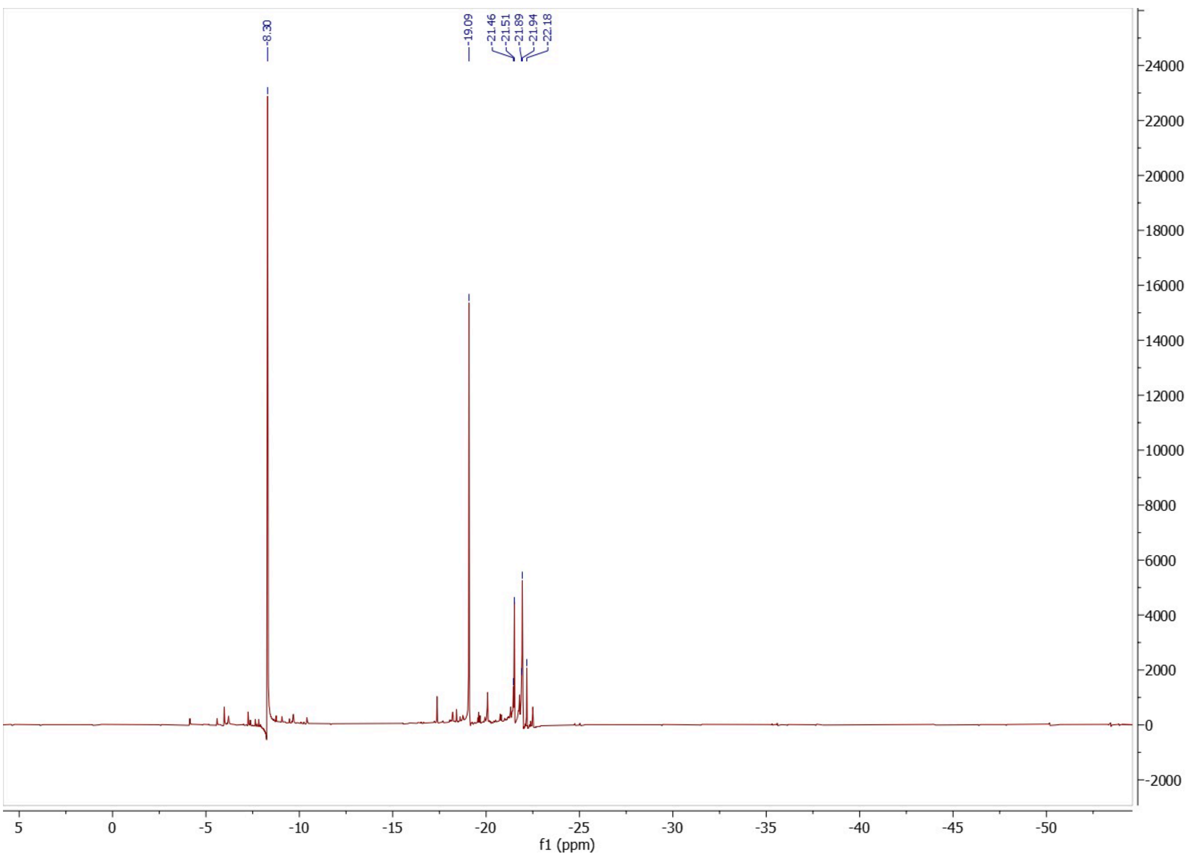

Figure S3: ^{29}Si-NMR of PTFE-V:SR-V mixture (1:1) liquid product

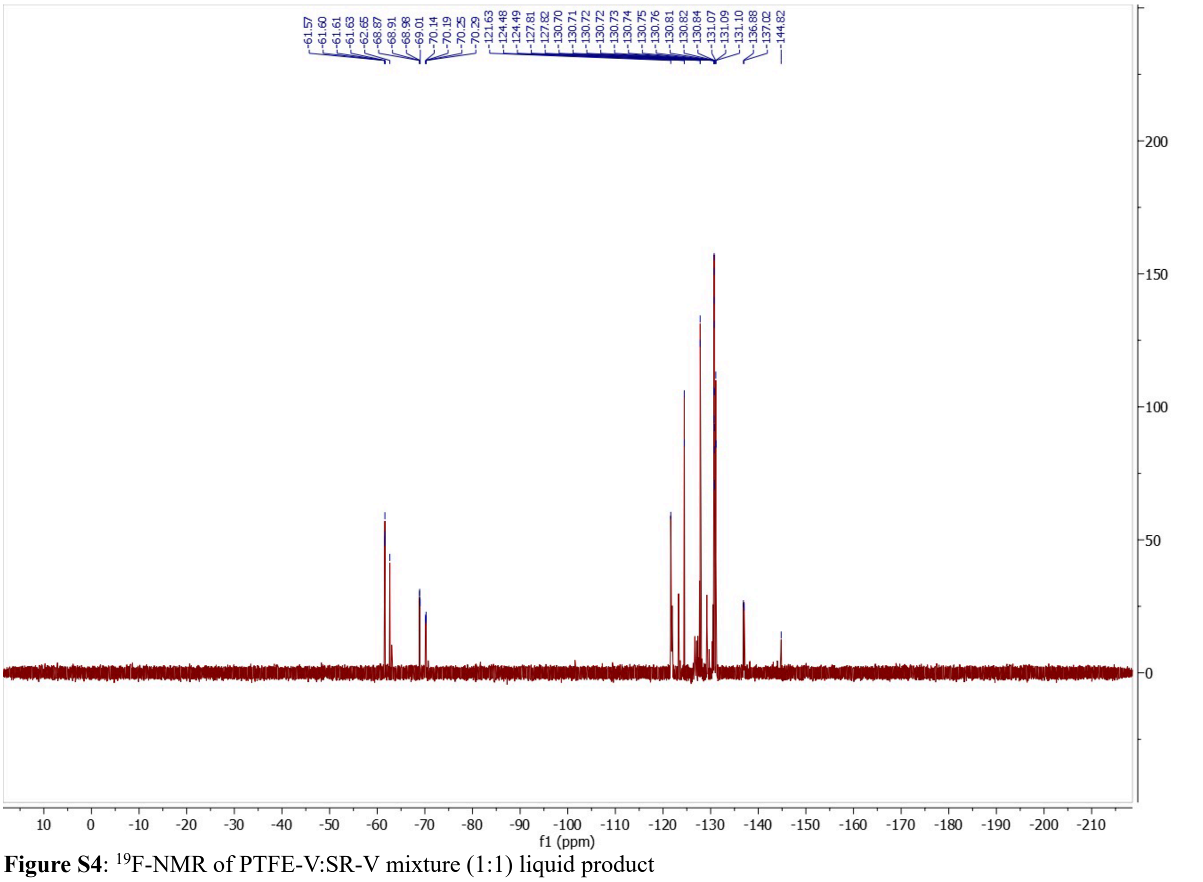

Figure S4: ^{19}F-NMR of PTFE-V:SR-V mixture (1:1) liquid product

Table S1: Individual mass-balance results of two independent experimental runs for the investigated polymer mixtures

PTFE-V content (wt%)	Gaseous (wt%)		Liquid & Solid (wt%)		Residue (wt%)	
	1.	2.	1.	2.	1.	2.
20	14.67	14.98	50.09	50.87	35.24	34.15
50	34.01	34.75	44.28	43.80	21.71	21.45
75	48.01	47.61	40.03	41.05	11.96	11.34
90	82.20	81.70	13.28	13.60	4.52	4.70
**PTFE-T content (wt%)**						
20	19.18	19.74	49.01	49.57	31.81	30.69
50	41.94	42.78	36.21	37.17	21.85	20.05

Table S2: Compounds identified in the gaseous products of PTFE-V, PTFE-V:SR-V and composites

#	Compound	m/z (% abundance)	RT (min)	Assignment
1	Methane	15 (85), 16 (100)	4.675	Confirmed via reference substance
2	Ethane	28 (8), 44 (100)	5.910	Confirmed via reference substance
3	Tetrafluoroethylene	31 (99), 50 (100), 81 (99), 100 (99)	6.459	Confirmed via literature data
4	Fluoroform	31 (33), 51 (100), 69 (35), 81 (14), 100 (9)	6.898	Library Match
5	1,1-Difluoroethylene	31 (27), 44 (39), 45 (55), 64 (100)	7.058	Library Match
6	Unidentified	25 (24), 26 (100), 32 (8)	8.291	unidentified
7	Trifluoroethylene	31 (39), 51 (45), 63 (85), 82 (100)	8.892	Library Match
8	Fluoroethylene	26 (37), 45 (45), 46 (100)	9.300	Library Match
9	Difluoromethane	33 (100), 51 (84)	9.422	Library Match
10	Fluoromethane	15 (15), 33 (81), 34 (100)	9.863	Library Match
11	Unidentified	51 (100), 69 (5), 101 (82)	11.168	unidentified
12	Octafluorocyclobutane	69 (39), 100 (45), 131 (100), 150 (58)	11.275	Confirmed via literature data
13	Unidentified	75 (50), 94 (90), 100 (16), 131 (100), 150 (61)	11.884	unidentified
14	Unidentified	51 (25), 77 (100), 78 (74), 131 (13), 150 (9)	12.435	unidentified
15	Hexafluoropropene	69 (7), 100 (100), 131 (97)	13.132	Confirmed via literature data
16	1,1,1,2-Tetrafluoroethane	69 (8), 83 (8) 100 (100), 131 (97)	13.519	Library Match
17	Pentafluoroprop-1-ene	69 (54), 82 (23), 93 (12), 100 (19), 113 (100), 132 (54)	13.685	Library Match
18	1,1,2,3,3-Pentafluoropropene	63 (21), 69 (92), 82 (100), 93 (17), 100 (45), 113 (93), 131 (53), 132 (97)	13.922	Library Match
19	Unidentified	51 (23), 75 (20), 77 (68), 95 (100), 96 (96), 100 (42), 131 (56)	14.143	unidentified
20	1,1,2,2-Tetrafluoroethane	33 (38), 51 (100), 63 (7), 83 (70), 101 (10), 131 (5)	14.366	Library Match

21	C_4F_8	51 (10), 69 (13), 82 (13), 93 (14), 100 (18), 113 (25), 131 (100), 150 (9), 181 (26), 200 (22)	14.475	Assignment via Reference Spectra from John Wiley & Sons
22	C_4F_8	93 (7) 131 (100), 181 (11), 200 (12)	14.706	Assignment via Reference Spectra from John Wiley & Sons
23	C_4F_8	51 (100), 69 (98), 100 (17), 119 (7), 151 (22)	14.843	Assignment via Reference Spectra from John Wiley & Sons
24	2-Fluoropropene	51 (16), 69 (100), 82 (27), 151 (42)	15.116	Library Match
25	Difluorodimethylsilane	47 (19), 81 (100), 96 (14)	15.220	Library Match
26	C_5F_{10}	69 (50), 93 (21), 100 (100), 131 (61), 150 (8), 181 (99), 200 (16), 231 (7)	15.864	Assignment via Reference Spectra from John Wiley & Sons
27	C_4F_{10}	69 (100), 75 (11), 95 (80), 113 (59), 119 (48), 131 (17), 163 (18), 169 (21), 181 (11)	16.460	Assignment via Reference Spectra from John Wiley & Sons
28	3,3,3-Trifluoropropene	46 (48), 51 (100), 77 (38), 95 (100), 96 (96)	17.049	Library Match
29	1,1,2,2,-Tetrafluoropropane	51 (31), 65 (100), 77 (54), 78 (24), 101 (11), 131 (46), 181 (15)	17.444	Library Match
30	1,1,1,2,3,3-Hexafluoropropane	51 (100), 64 (12), 69 (29), 82 (43), 133 (15)	17.738	Library Match
31	Unidentified	51 (100), 69 (66), 95 (24), 101 (13), 113 (16), 119 (13), 131 (12), 145 (19), 164 (8)	17.839	unidentified
32	Pentafluoroethane	51 (100), 69 (31), 82 (74), 101 (33), 113 (3), 133 (3), 151 (1)	18.206	Library Match
33	Decafluoropentene	69 (100), 100 (35), 119 (41), 131 (23), 169 (42), 219 (13), 231 (9), 243 (9), 262 (4)	18.973	Tenative, assignment via MS interpretation
34	C_5F_{10}	93 (49), 131 (21), 143 (34), 162 (100), 193 (33), 212 (11), 243 (67), 262 (22)	19.057	Tenative, assignment via MS interpretation
35	Unidentified	51 (100), 69 (53), 101 (21), 119 (18), 131 (16), 151 (3), 163 (5), 181 (7), 200 (7)	20.284	unidentified
36	Unidentified	75 (74), 94 (18), 125 (22), 144 (100), 175 (59), 194 (52)	20.434	unidentified
37	Octafluorohexene	51 (100), 69 (61), 75 (19), 95 (21), 101 (31), 113 (40), 132 (30), 144 (22), 151 (19), 175 (14), 194 (12)	20.551	Library Match
38	C_6F_{12}	43 (100), 57 (60), 75 (18), 131 (69), 144 (24), 163 (11), 175 (11), 214 (5), 231 (5), 243 (2)	21.056	Tenative, assignment via MS interpretation
39	Trimethylfluorosilane	47 (28), 49 (15), 63 (8), 77 (100), 92 (6)	21.228	Library Match
40	C_6F_{14}	43 (100), 51 (83), 57 (53), 69 (60), 119 (41), 131 (38),	23.245	Assignment via Reference Spectra from John Wiley & Sons

		169 (37), 176 (19), 219 (23), 269 (9)		
41	Unidentified	51 (100), 69 (29), 101 (29), 113 (13), 131 (22), 151 (6), 163 (14), 181 (12)	23.888	unidentified
42	Unidentified	51 (100), 69 (29), 101 (27), 131 (27), 151 (27), 151 (15), 213 (8), 231 (5)	27.445	unidentified

Table S3: Fragmentation pattern, normalized peak area (100%) and possible structure of PTFE-V:SR-V liquid products at varying PTFE-V content determined via GC-MS.

**Product Group**	**Possible Structure**	**m/z (% abundance)**	**RT (min)**	**25% SR-V**	**50% SR-V**	**80% SR-V**	**100% SR-V**	**Assignment**
				**Normalized Peak Area (100%) (%)**				
C_xF_{2x+1}	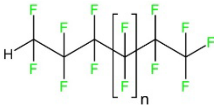 n = 0	51 (100), 69 (46), 101 (29), 131 (32), 151 (16), 181 (5), 201 (5), 231 (3), 251 (1)	2.277	1.99	1.52	<0.1	<0.1	Assignment via fragmentation pattern of other reported C_xF_{2x+1} substances
C_xF_{2x+1}	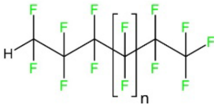 n = 1	51 (100), 69 (50), 101 (28), 131 (34), 153 (35), 181 (7), 201 (5), 233 (11), 281 (1), 301 (1)	2.780	2.21	3.65	0.22	<0.1	Assignment via reference spectrum from John Wiley and Sons Library
D_xF	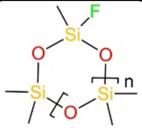 n = 1	98 (14), 137 (9), 167 (4), 195 (7) 211 (100), 212 (20), 213 (12), 214 (2)	3.052	1.94	4.74	2.90	<0.1	Tentative, derived from D_3 MS-spectrum
L_xF_2	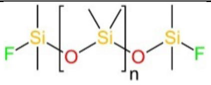 n = 1	73 (100), 107 (12), 155 (8), 193 (5), 213 (5), 229 (79), 230 (20), 231 (12)	3.287	4.88	4.91	1.74	<0.1	Tentative, derived from L_3 MS-spectrum
C_xF_{2x+1}	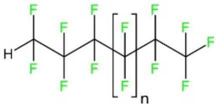 n = 2	51 (100), 69 (46), 101 (25), 131 (33), 151 (17), 181 (7), 201 (5), 231 (4), 281 (2), 331 (1)	3.344	1.87	1.10	0.07	<0.1	Assignment via reference spectrum from John Wiley and Sons Library
D_x	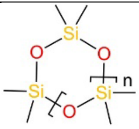 n = 1	96 (28), 133 (11), 177 (5), 191 (17), 207 (100), 208 (31), 209 (19), 210 (3)	3.481	9.74	16.77	47.52	64.47	Confirmed via reference material
U_x	unidentified	99 (25), 141 (7), 185 (7), 215 (100, 216 (20), 217 (11), 259 (19), 260 (3)	3.640	0.38	0.81	<0.1	<0.1	unidentified
U_x	unidentified	99 (22), 141 (6), 185 (7), 215 (100), 216 (19), 217 (11), 259 (14), 260 (3)	3.725	0.26	0.95	<0.1	<0.1	unidentified
-	L_3F_3	73 (61), 77 (27), 109 (6), 137 (8), 155 (7), 181 (6), 197 (4), 217 (5), 233 (100)	3.776	0.40	0.33	<0.1	<0.1	Tentative, derived from L_3 MS-spectrum
C_xF_{2x+1}	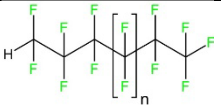 n = 3	51 (100), 69 (51), 101 (27), 131 (36), 151 (18), 181 (8), 201 (5), 231 (5), 281 (3), 301 (1), 331 (1), 381 (1)	3.889	1.34	0.77	0.07	<0.1	Assignment via fragmentation pattern of other reported C_xF_{2x+1} substances
U_x	unidentified	99 (14), 137 (7), 167 (4), 195 (8), 211 (100), 212 (22), 213 (14), 255 (24), 256 (5)	4.154	4.87	5.02	1.67	<0.1	unidentified

D_xF_2	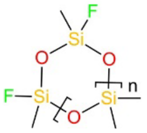 n = 2, fluorine position not explicit	73 (10), 137 (23), 181 (10), 215 (11), 273 (9), 289 (100), 290 (26), 291 (17) 292 (3)	4.304	0.55	0.77	0.02	<0.1	Tentative, derived from D_4 MS-spectrum
C_xF_{2x+1}	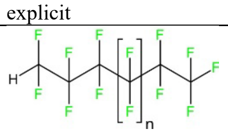 n = 4	51 (100), 69 (59), 101 (33), 131 (45), 151 (24), 181 (12), 201 (7), 231 (8), 281 (3), 331 (2), 381 (1), 431 (1)	4.450	2.13	0.82	0.08	<0.1	Assignment via fragmentation pattern of other reported C_xF_{2x+1} substances
-	L_4F_3	73 (39), 137 (12), 211 (100), 212 (21), 213 (15), 289 (49), 290 (12), 291 (9), 307 (9)	4.521	1.16	1.11	0.15	<0.1	Tentative, derived from L_4 MS-spectrum
-	unidentified	73 (10), 137 (23), 215 (13), 231 (8), 273 (9), 289 (100), 290 (27), 291 (17), 292 (2)	4.593	0.23	0.17	<0.1	<0.1	unidentified
-	unidentified	73 (5), 98 (10), 137 (8), 191 (6), 207 (14), 211 (100), 212 (18), 213 (11), 227 (21), 228 (4), 289 (3)	4.732	2.79	1.21	<0.1	<0.1	unidentified
D_xF_2	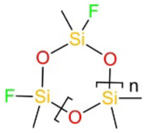 n = 2, fluorine position not explicit	73 (15), 99 (21), 137 (24), 215 (58), 259 815), 273 (11), 289 (100), 290 (25), 291 (16), 292 (3)	4.797	0.42	0.80	<0.1	<0.1	Tentative, derived from D_4 MS-spectrum
C_xF_{2x+1}	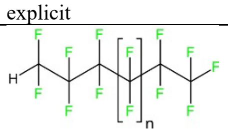 n = 5	51 (100), 69 (58), 101 (29), 131 (42), 151 (24), 181 (12), 201 (6), 231 (7), 251 (1), 281 (3), 331 (2), 431 (1), 481 (1)	5.001	1.08	0.55	0.11	<0.1	Assignment via fragmentation pattern of other reported C_xF_{2x+1} substances
D_xF	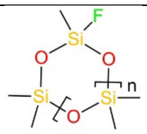 n = 2	73 (6), 135 (11), 193 (6), 211 (5), 269 (12), 285 (100), 286 (28), 287 (17), 288 (3)	5.048	6.38	4.36	3.47	<0.1	Tentative, derived from D_4 MS-spectrum
L_xF_2	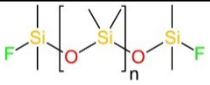 n = 2	73 (100), 207 (10), 211 (24), 303 (12), 304 (3), 305 (2)	5.365	4.20	4.20	0.79	<0.1	Tentative, derived from L_4 MS-spectrum
D_x	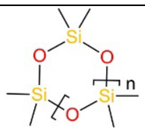 n = 2	73 (6), 133 (14), 193 (8), 249 (11), 265 (16), 281 (100), 282 (37), 283 (23), 284 (5)	5.392	11.78	11.51	24.09	20.97	Confirmed via reference material
D_xF_2	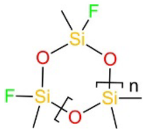 n = 2, fluorine position not explicit	73 (23), 99 (100), 137 (12), 215 (17), 237 (17), 273 (10), 289 (97), 290 (26), 291 (17), 292 (3)	5.460	0.46	0.37	<0.1	<0.1	Tentative, derived from D_4 MS-spectrum
D_xF_2		73 (10), 99 (19), 137 (12), 193 (5), 215 (9), 273 (7), 289 (100),	5.525	2.61	1.26	<0.1	<0.1	

		290 (25), 291 (17), 292 (3)						
D_xF_2		99, 137 (14), 215 (8), 273 (8), 289 (100), 290 (27), 291 (18), 292 (3)	5.569	1.52	0.76	<0.1	<0.1	
U_x	unidentified	73 (10), 99 (12), 135 (11), 193 (6), 211 (5), 269 (9), 285 (100), 286 (27), 287 (19), 288 (4), 329 (1)	5.895	5.12	3.34	0.98	<0.1	unidentified
D_xF_2	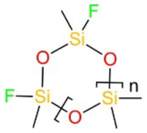	73 (100), 151 (7), 271 (41), 289 (8), 363 (11), 364 (4), 365 (3)	6.426	0.69	0.82	<0.1	<0.1	Tentative, derived from D_5 MS-spectrum
D_xF_2	n = 3, fluorine position not explicit	73 (100), 151 (6), 271 (44), 363 (12), 364 (4), 365 (3)	6.508	0.50	0.49	<0.1	<0.1	
D_xF	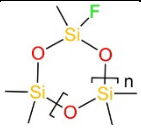 n = 3	73 (100), 267 (71), 359 (41), 360 (14), 361 (10), 362 (2)	6.739	4.24	3.39	1.01	<0.1	Tentative, derived from D_5 MS-spectrum
D_x	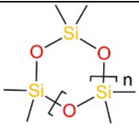 n = 3	73 (100), 267 (46), 355 (67), 356 (25), 357 (16), 358 (4)	7.045	6.35	5.16	8.58	14.77	Confirmed via reference material
L_xF_2	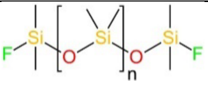 n = 3	73 (81), 151 (29), 207 (20), 271 (14), 285 (100), 286 (27), 287 (16), 288 (7), 363 (8), 377 (8)	7.171	2.30	1.84	0.28	<0.1	Tentative, derived from L_5 MS-spectrum
D_xF	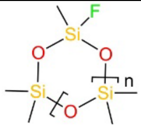 n = 4	73 (100), 267 (50), 359 (33), 360 (11), 361 (7), 403 (3)	7.470	1.46	0.79	0.15	<0.1	Tentative, derived from D_6 MS-spectrum
D_xF_2	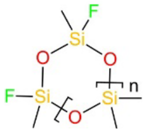	73 (100), 329 (6), 345 (40), 437 (3)	7.939	0.23	0.18	0.1	<0.1	Tentative, derived from D_6 MS-spectrum
D_xF_2	n = 4, fluorine position not explicit	73 (100), 329 (4), 345 (24), 437 (1)	8.069	0.53	0.80	<0.1	<0.1	
D_xF_2		73 (100), 329 (7), 345 (44), 437 (3)	8.137	0.18	0.19	<0.1	<0.1	
D_xF	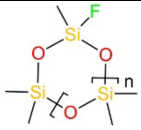 n = 5	73 (100), 325 (14), 341 (67), 342 (24), 343 (17), 433 (7)	8.405	0.95	0.54	0.12	<0.1	Tentative, derived from D_7 MS-spectrum
D_x	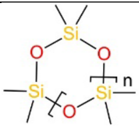 n = 5	73 (100), 147 (20), 325 (14), 341 (55), 342 (17), 343 (12), 429 (27), 430 (12), 431 (8)	8.698	2.80	3.00	4.43	6.06	Confirmed via literature data and database
L_xF_2	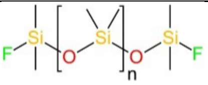 n = 4	73 (100), 147 (16), 225 (77), 267 (23), 281 (24), 285 (15), 359 (10) 451 (1)	8.752	2.08	1.34	0.1	<0.1	Tentative, derived from L_6 MS-spectrum
U_x	unidentified	73 (100), 151 (8), 229 (8), 285 (10), 341 (48),	9.024	0.50	0.37	<0.1	<0.1	unidentified

		342 (15), 343 (10), 433 (4), 477 (2)						
-	unidentified	73 (100), 225 (14), 267 (24), 281 (23), 341 (20), 355 (40), 449 (1)	9.752	<0.1	0.38	<0.1	<0.1	unidentified
D_xF	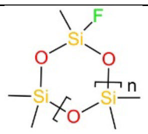 n = 6	73 (100), 147 (13), 281 (28), 327 (21), 341 (9), 399 (2), 415 (8), 507 (1)	9.895	0.21	0.11	<0.1	<0.1	Tentative, derived from D_8 MS-spectrum
L_xF_2	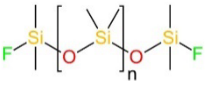 n = 5	73 (100), 147 (13), 151 (9), 207 (22), 225 (70), 267 (8), 281 (12), 299 (9), 303 (6), 325 (4), 341 (13), 359 (5), 525 (<1)	10.143	1.29	1.15	0.44	<0.1	Tentative derived from typical fragmentation pattern of L_xF_2 fraction and periodic retention time spacing
D_x	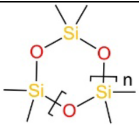 n = 6	73 (100), 147 (43), 221 (8), 281 (48), 327 (16), 341 (7), 383 (2), 399 (3), 415 (8), 503 (6)	10.188	0.82	1.64	1.03	2.28	Confirmed via literature data and database
L_xF_2	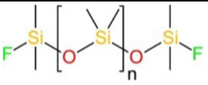 n = 6	73 (100), 147 (18), 207 (30), 225 (38), 281 (14), 299 (19), 341 (9), 377 (3), 415 (2) 429 (1), 599 (<1)	11.385	1.15	0.93	<0.1	<0.1	Tentative derived from typical fragmentation pattern of L_xF_2 fraction and periodic retention time spacing
D_x	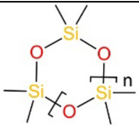 n = 7	73 (100), 147 (26), 221 (18), 281 (13), 355 (27), 401 (5), 416 (1), 488 (<1) 577 (<1)	11.516	0.35	1.33	<0.1	<0.1	Confirmed via literature data and database
L_xF_2	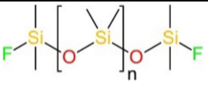 n = 7	73 (100), 147 (25), 207 (29), 225 (51), 281 (31), 299 (19), 373 (4), 401 (3), 429 (2), 451 (1), 503 (<1), 673 (<1)	12.507	1.02	0.83	<0.1	<0.1	Tentative derived from typical fragmentation pattern of L_xF_2 fraction and periodic retention time spacing
D_x	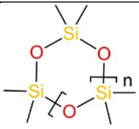 n = 8	73 (100), 147 (24), 221 (13), 281 (6), 355 (7), 429 (7), 475 (1), 651 (<1)	12.660	0.32	0.70	<0.1	<0.1	Confirmed via literature data and database
L_xF_2	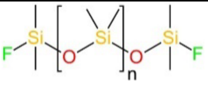 n = 8	73 (100), 147 (25), 207 (29), 225 (51), 281 (31), 299 (19), 373 (4), 401 (3), 429 (2), 451 (1), 503 (<1), 747 (<1)	13.529	0.66	0.63	<0.1	<0.1	Tentative derived from typical fragmentation pattern of L_xF_2 fraction and periodic retention time spacing
D_x	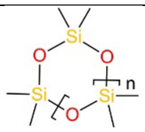 n = 9	73 (100), 147 (27), 221 (11), 281 (18), 355 (7), 429 (2), 503 (1), 725 (<1)	13.677	0.08	0.25	<0.1	<0.1	Confirmed via literature data and database

L_xF_2	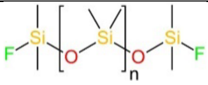 n = 9	73 (100), 147 (25), 207 (29), 225 (51), 281 (31), 299 (19), 373 (4), 401 (3), 429 (1), 451 (1), 821 (<1)	14.464	0.43	0.50	<0.1	<0.1	Tentative derived from typical fragmentation pattern of L_xF_2 fraction and periodic retention time spacing
D_x	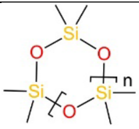 n = 10	73 (100), 147 (27), 221 (11), 281 (18), 355 (7), 429 (2), 503 (1), 799 (<1)	14.609	0.05	0.15	<0.1	<0.1	Confirmed via literature data and database
L_xF_2	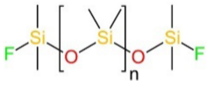 n = 9	73 (100), 147 (25), 207 (29), 225 (51), 281 (31), 299 (19), 373 (4), 401 (3), 429 (1), 451 (1)	15.330	0.33	0.39	<0.1	<0.1	Tentative derived from typical fragmentation pattern of L_xF_2 fraction and periodic retention time spacing
L_xF_2	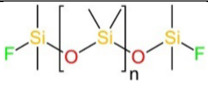 n = 10	73 (100), 147 (25), 207 (29), 225 (51), 281 (31), 299 (19), 373 (4), 401 (3), 429 (1), 451 (1)	16.133	0.15	0.29	<0.1	<0.1	Tentative derived from typical fragmentation pattern of L_xF_2 fraction and periodic retention time spacing

Table S4: Relative abundance of different product groups determined by normalized GC–MS peak areas of different pyrolysis experiments for PTFE-V:SR-V mixtures and composite materials

	**90 wt% PTFE-V**	**75 wt% PTFE-V**	**50 wt% PTFE-V**		**20% PTFE-V**		**PTFE-SR-T**			**PTFE-SR-Septum**		
	**1.**	**1.**	**1.**	**2.**	**1.**	**2.**	**1.**	**2.**	**3.**	**1.**	**2.**	**3.**
D_x	11.6	32.3	40.5	41.8	85.7	86.6	93.9	94.0	94.0	62.5	61.8	63.0
D_xF	6.4	15.2	13.9	16.7	7.5	6.1	3.2	3.5	3.1	17.6	17.2	16.7
D_xF_2	22.3	7.7	6.4	5.0	0.1	0.2	0.3	0.3	0.2	2.0	2.0	2.1
L_xF_2	6.1	18.5	17.0	16.3	3.4	5.3	2.0	1.9	2.2	9.5	9.6	9.3
C_xF_{2x+1}	22.2	10.6	8.4	8.7	0.6	0.5	<0.01	<0.01	<0.01	1.6	2.1	2.0
U_x	8.8	11.1	10.5	7.5	2.7	1.4	0.6	0.3	0.4	3.5	3.7	3.6
Other	22.7	4.6	3.2	3.8	0.2	0.1	0.1	0.1	0.1	3.3	3.6	3.3
